# Synapse-Mimicking Memristors Based on 3,6-Di(*tpy*)-9-Phenylcarbazole Unimer and Its Copolymer with Cobalt(II) Ions

**DOI:** 10.3390/polym16040542

**Published:** 2024-02-17

**Authors:** Ambika Pandey, Andrei Chernyshev, Yadu Ram Panthi, Jiří Zedník, Adriana Šturcová, Magdalena Konefał, Olga Kočková, Stephen H. Foulger, Jiří Vohlídal, Jiří Pfleger

**Affiliations:** 1 Faculty of Mathematics and Physics, Charles University, Ke Karlovu 3, 121 16 Prague, Czech Republic; pandey@imc.cas.cz (A.P.); panthi@imc.cas.cz (Y.R.P.); 2 Institute of Macromolecular Chemistry, Czech Academy of Sciences, Heyrovského nám. 2, 162 00 Prague, Czech Republic; sturcova@imc.cas.cz (A.Š.); magdalenakonefal@imc.cas.cz (M.K.); kockova@imc.cas.cz (O.K.); 3 Department of Physical and Macromolecular Chemistry, Faculty of Science, Charles University, Albertov 6, 128 00 Prague, Czech Republic; andrei.chernyshev@natur.cuni.cz (A.C.); jiri.zednik@natur.cuni.cz (J.Z.); 4 Center for Optical Materials Science and Engineering Technology (COMSET), Department of Materials Science and Engineering, Clemson University, Clemson, SC 29634, USA; foulger@clemson.edu; 5 Department of Bioengineering, Clemson University, Clemson, SC 29634, USA

**Keywords:** carbazole, coordination polymer, memristor, metallo-supramolecular polymer, synapse-mimicking device, 2,2′:6′,2″-terpyridine

## Abstract

The title compound, unimer **U** (*tpy* stands for 2,2′:6′,2″-terpyridin-4′-yl end-group), by itself shows the memristor effect with a retention time of 18 h and persistence of 11 h. Its coordination copolymer with Co(II) ions, **[CoU]_n_**, exhibits multimodal resistance changes similar to the synaptic responses observed in biological systems. More than 320 cycles of potentiation and depression measured in continuous sequence occurred without observing a significant current change, confirming the operational stability and reproducibility of the device based on the **[CoU]_n_** polymer. The synaptic effect of a device with an indium tin oxide (ITO)/**[CoU]_n_**/top-electrode (TE) configuration is more pronounced for the device with TE = Au compared to devices with TE = Al or Ga. However, the latter TEs provide a cost-effective approach without any significant compromise in device plasticity. The detected changes in the synaptic weight, about 12% for pair-pulse facilitation and 80% for its depression, together with a millisecond trigger and reading pulses that decay exponentially on the time scale typical of neurosynapses, justify the device’s ability to learn and memorize. These properties offer potential applications in neuromorphic computation and brain-inspired synaptic devices.

## 1. Introduction

The human brain has an enormous memory and incredible processing capabilities based on its special operational skills such as parallel processing and event-driven operations. For years, researchers have been trying to develop a device that can mimic the functionality of the human brain. The development of memristors can lead to the fulfillment of these needs. A memristor is an electronic element with a resistance dependent on the charge that has previously passed through it. The concept and mathematical model of the memristor as the fourth basic passive component of electronic circuits were introduced by Leon Chua in 1971. A functional prototype of the memristor was presented by HP Lab in 2008, and since then, research has been increasingly focused on direct applications of memristors in current electronic circuits [1,2].

First, the memristor principles have been anticipated to be exploited in various memory devices within a classical von Neumann computer architecture, like in dynamic random-access memory (DRAM), static random-access memory (SRAM), flash and write-once-read-many (WORM) memory. The properties of memristors were found to be particularly suitable for resistive random-access memories (ReRAM) [2,3]. In this scenario, an abrupt current increment/decrement at a certain applied voltage creates two conducting states, high-resistance (HR) and low-resistance (LR) states, representing the logical “0” and “1” in digital electronics [4]. However, conventional systems constrained by the von Neumann architecture based on the binary logic have adverse limitations for advanced computing methods like neuromorphic computing. Unlike binary logic, neuromorphic computing requires an electronic element with continuously varying states depending on previous input signals. The input signal consists of a sequence of voltage spikes that stimulate the device, similar to what a neural synapse performs in biological systems. These trains of spikes change the output state of the device continuously to the LR state, mimicking a learning process. When no signal or a signal of the opposite polarity appears, the memory state is gradually erased, thereby mimicking the process of forgetting [5]. The device should possess several important synaptic functionalities, such as memorizing, memory loss, and memory transition. Due to these features, memristors are considered as possible building blocks of brain-inspired neuromorphic computers and artificial neural networks [5].

Significant effort has been invested in the development of novel functional memristive materials [2,6,7,8]. Initially, inorganic materials dominated in this field, but recently, their organic low-molecular and macromolecular counterparts have gained increasing attention due to their lower cost and easier processing [4,9,10,11]. So far, mainly various azole compounds have been used due to their ReRAM functionality that is usable for data storage [4,10,11]. Among the perspective materials are carbazole derivatives, who’s layers usually show good charge transport properties facilitated by a strong exchange integral between adjacent molecules in a face-to-face arrangement [12,13]. Materials composed of molecules with both electron-donating and electron-accepting groups (D-A materials) are even more attractive for organic electronics due to their remarkable properties stemming from the cumulative charge transfer (CT) [13,14,15]. Yang Yu et al. reported a synaptic memristive effect for D-A-type conjugated macrocycles referred to as gridarenes [16] that form solid films with nanopores that facilitate the transport of ions, which is responsible for the observed memristive functionality.

Conjugated coordination compounds and metallo-supramolecular polymers represent relatively new classes of materials for modern electronics. They often possess a reasonable stability of electronic states and a narrow bandgap, enabling resistive switching with low energy consumption. The observed good reproducibility of their memristive characteristics is attributed to the interplay of charge transfer with redox processes [9,17,18,19]. However, depending on the nature of the functional materials and on the device structure, the memristive effect can also be based on the conformational changes in the molecules or interfacial phenomena induced by the electric field [9,20,21]. S. Goswami et al., who studied the memristive and neuromorphic properties of 2-(phenylazo)pyridine ruthenium(II) ions, demonstrated that the redox state of the ligand determines the switching states of the memristive device, while the counterions control the hysteresis of the device [9].

π-Conjugated compounds with two or more *tpy* end-groups have been in focus for more than two decades as they enable the construction of 2D and 3D metallo-supramolecular polymers applicable in the fields such as catalysis, sensing, photovoltaics, electrochromism, and redox processes [22,23,24,25,26,27]. Xi Yang et al. reported synaptic memristor plasticity for di(*tpy*)-1,4-phenylene assembled with Fe^2+^ ions [28].

In the present work, we report memristive and synapse-mimicking effects disclosed for devices with active layers from a bis(tpy)carbazole ditopic ligand, hereinafter referred to as unimer **U**, and a supramolecular polymer formed by the self-assembly of **U** with cobalt Co^2+^ ions, hereinafter referred to as **[CoU]_n_**. The memristor with a **U** active layer exhibits an excellent nonvolatile bistable memory behavior, while that with a **[CoU]_n_** layer exhibits both bistable memory and synaptic plasticity.

## 2. Materials and Methods

### 2.1. Materials

4′-Bromo-2,2′:5′,2″-terpyridine (TCI, Tokyo, Japan), 3,6-bis(4,4,5,5-tetramethyl-1,3,2-dioxaborolan-2-yl)-9-phenylcarbazole (ABCR, Karlsruhe, Germany), 1,1,1,3,3,3-hexafluoropropan-2-ol (Fluorochem, Hadfield, UK), dimethylformamide (DMF), acetone, acetonitrile, hexane, methanol, propan-2-ol, propylene carbonate, tetrabutylammonium hexafluorophosphate (NBu_4_PF_6_), and cobalt (II) perchlorate hexahydrate (all solvents of analytical grade, Sigma-Aldrich and Merck, Prague, Czech Republic) were used as-received. Tetrakis(triphenylphosphine)palladium(0) (Pd(PPh_3_)_4_ (Merck, Prague, Czech Republic) was washed three times with methanol (20 mL) and dried before use. Quartz substrates used for optical spectroscopy measurement were purchased from Präzisions Glas & Optik GmbH (Iserlohn, Germany). ITO-coated glass substrates with dimensions of 15 × 20 mm used for thin film preparation for the electrical and electrochemical measurements and the electrode setup required for the electrochemical measurement were purchased from Ossila BV (Leiden, The Netherland). Silicon oxide substrates were used for GIWAX and were purchased from Ossila BV (The Netherland).

### 2.2. Instrumentation

NMR spectra were recorded on a Varian Unity Inova 400 MHz spectrometer in 1,1,2,2-tetrachloroethylene-*d*_2_ (TCE-*d*_2_) at 110 °C (owing to the limited solubility of **U**) and referenced to the solvent signal (6 ppm for ^1^H and 64 ppm for ^13^C spectra). IR spectra were measured using a Nicolet Nexus 870 FTIR instrument (Thermo Fisher Scientific, Waltham, MA, USA) equipped with a Vari GATRTM attachment (Harrick Scientific Products, Inc., Pleasantville, NY, USA, incidence angle ranging from 60° to 65°) and a horizontal micro-ATR Golden Gate unit SPECAC (256 scans resolution of 4 cm^−1^). UV/vis spectra were measured on a PerkinElmer Lambda 950 (Waltham, MA**,** USA) spectrometer and luminescene spectra on an FS5 spectrofluorimeter (Edinburg Instruments, Livingston, Edinburgh, UK). MALDI-TOF mass spectra were acquired with an UltrafleXtreme TOF–TOF mass spectrometer (Bruker Daltonics, Bremen, Germany) equipped with a 2000 Hz smartbeam-II laser (355 nm) using the positive ion linear/reflectron mode. Panoramic pulsed ion extraction and external calibration were used for molecular weight assignment. Roughness and thickness profiles of the prepared layers were obtained from 2D and 3D surface scans (speed of 2 µm/s, frequency of 2000 Hz, force constant of 0.5) using a KLA TENCOR P-17 surface profilometer (KLA-Tencor, Milpitas, CA, USA). Powder XRD (X-ray diffraction) was measured at room temperature on a high-resolution Explorer diffractometer (GNR, Agrate Conturbia, Italy) equipped with a Mythen 1K silicon-strip detector using the Bragg–Brentano geometry, CuKα radiation (1.54 Å), and a 2*θ* range of 2–50° with a step of 0.05° and a time of 15 s in each step. Grazing incidence wide-angle X-ray scattering (GIWAXS) measurements were performed using a pinhole camera (MolMet, Rigaku, Tokyo, Japan, upgraded by SAXSLAB/Xenocs, Grenoble, France) equipped with a vacuum version of the Pilatus 300 K detector and attached to a micro-focused X-ray beam generator (Rigaku MicroMax 003, Tokyo, Japan) operating at 0.6 mA and 50 kV. The sample-to-detector distance was 211 mm, the incident scattering angle α_inc_ was 0.19°, and the exposure time of each measurement was 6 h. The electrical characteristics of the prepared devices were measured on a Keithley 2602 dual-channel source meter (Keithley Instruments, Solon, OH, USA) using a serial connection of the device, power supply, and source meter. Cyclic voltammetry was measured on an AMEL s.r.l. Potentiostat (AMEL, Milano, Italy) using thin films (~200 nm) of active materials deposited on ITO as the working electrode, an acetonitrile solution of n-Bu_4_NPF_6_ (0.1 M) as the electrolyte, an Ag/AgCl reference electrode, a Pt wire counter electrode, and ferrocene as an external reference. For spectro-electrochemical measurements, the following sandwich structures were prepared: | cathode | thin working material film | gel electrolyte | anode |, where the thickness of the gel electrolyte layer was defined by a 150 nm spacer. The gel electrolyte solution was prepared by gradual addition of LiClO_4_ (10 g) and PMMA (10 g) into propylene carbonate (55 mL) under stirring (transparent solution was obtained after 5 h of stirring).

More details on the instrumentation used can be found in the Supplementary Materials (SM).

## 3. Procedures

**Scheme 1:**
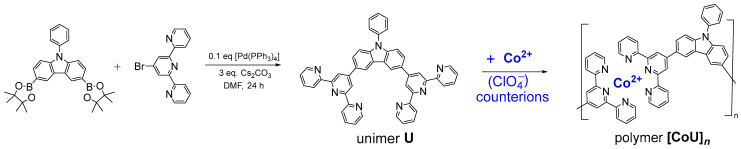
Synthesis of unimer **U** and its transformation to organometallic (metallo-supramolecular) copolymer **[CoU]_n_** (note that the planes of *tpy* groups coordinated to a Co^2+^ ion are perpendicular to each other).

**Unimer U**: 3,6-di(2,2′:6′,2″-terpyridin-4′-yl)-9-phenylcarbazole was prepared by means of Suzuki coupling (Scheme 1). 4′-Bromo-2,2′:6′,2″-terpyridine (350 mg, 1.22 mmol, 2 equivalents) was added to a solution of 3,6-bis(4,4,5,5-tetramethyl-1,3,2-dioxaborolan-2-yl)-9-phenylcarbazole (280 mg, 0.56 mmol of 1 eq) in DMF (10 mL), and the resulting solution was bubbled with argon for 10 min, and fresh Pd(PPh_3_)_4_ (65 mg, 0.1 eq) and 1 g of Cs_2_CO_3_ (3 mmol) were added. The mixture was again bubbled with argon for 10 min and then heated and kept under stirring at 100 °C for 24 h. The sediment obtained after cooling the mixture in a freezer was filtered, washed gradually with distilled water (3 × 25 mL), hexane (3 × 25 mL), and methanol (3 × 25 mL) and dried overnight. The isolated yield was 286 mg (72%); melting at 314–315 °C. MALDI-TOF mass spectra: found *m*/*z*: 706.28; theory for C_48_H_31_N_7_: 706.27 (see Figure S1 in SM). For the NMR spectra and their assignment, see Figure S2; for the Raman spectra, see Figure S3; and for the IR spectra, see Figure S4.

**Polymer [CoU]_n_** was prepared by the self-assembling of unimer **U** with Co^2+^ ions, which was performed by mixing equimolar amounts (0.003 M) of **U** and Co(ClO_4_)_2_ in HFP/ACN mixed solvent (4/1 by volume) and stirring the solution overnight at room temperature. The assembly of **U** molecules with Co^2+^ ions into alternating macromolecules proceeded through the coordination of the 2,2′:6′,2″-terpyridin-4′-yl (*tpy*) end-groups to the ions, giving [*-*tpy-Co-tpy*-*] complex linkages with an octahedral geometry, in which planes of coordinated *tpy* groups were perpendicular to each other (axial vs equatorial orientation).

**Deposition of materials on substrates.** Before deposition, each substrate was washed in an ultrasonic bath successively with Mucasol alkaline liquid cleaning agent (Merck), deionized water, acetone, and propan-2-ol (for 15 min each), dried with nitrogen, and finally cleaned with ozone for 20 min. The surface resistance of the cleaned ITO was 30 Ω/cm^2^. Thin films of unimer **U** (5–300 nm) were deposited by physical vapor deposition (PVD) using a MiniLab 60 vacuum evaporator (Moorfield Nanotechnology Ltd., Knutsford, Cheshire, UK), a pressure of 10^−5^ Pa, a constant deposition rate of 0.3 Å/s, and a slowly rotating substrate (10 rpm). Scans of the prepared **U** layers (Figure S5 in SM) showed relatively smooth surfaces with a mean roughness of 10 nm. The **[CoU]_n_** polymer films (20–800 nm) and a few films of unimer **U** for comparison were deposited on substrates (glass, silica, ITO) by casting from the HFP/ACN solutions (2 mg/mL), in which a drop (100 μL) of the solution was spread over the entire surface of the substrate. The layers prepared by the drop-casting technique showed an average roughness of 40 nm (Figure S5).

**The memristive devices** prepared had the following sandwich architecture: ITO bottom electrode (BE)|**U** or **[CoU]_n_** active layer|Al, or Au, or Ga top electrode (TE, thickness 60–80 nm, see Figures S6 and S7). The Au and Al TEs were deposited by the PVD method (10^−5^ Pa, deposition rate of 5–10 Å/s) using a shadow mask. The gallium TE was deposited by placing a small piece of Ga heated to 30 °C at the desired position on the active layer, where it was allowed to cool to room temperature. All electrical measurements of the memristive devices were performed in ambient conditions (humidity 40–45%) at room temperature.

## 4. Results

### 4.1. Characterization of Studied Materials

The TGA analysis of the powdered **U** showed no mass loss at around 100 °C, which indicates that this material does not significantly adsorb moisture (Figure S8 in SM). The first signs of instability appeared at 430 °C, and a progressive decomposition of **U** occurred above 470 °C. This demonstrates its unusually high thermal stability, in which **U** significantly exceeds the vast majority of known bis(tpy) unimers, which decompose below 405 °C [29]. Moreover, it indicates that **U** does not change by its chemical composition during vacuum sublimation deposition onto substrates at temperatures between 220 °C and 270 °C. The invariability of the chemical nature of **U** after its deposition on the surface was confirmed by the matches of the Raman and IR vibrational spectra (Figures S3 and S4) and the excitation and emission optical spectra of the fresh and sublimation-deposited unimer **U** (Figure S9). The TGA record for **[CoU]_n_**, also shown in Figure S8, showed the onset of weight loss at 127 °C and a major weight loss starting above 150 °C, which can be attributed to the oxidation of organic components by perchlorate anions. The decomposition temperature is well above the working temperature of the prepared devices. Perchlorate counterions were used because, due to their low charge density associated with their bulkiness, they interact only slightly with the cationic centers of the **[CoU]_n_** chains, so they can easily diffuse and/or drift in the polymer [30,31].

**Figure 1:**
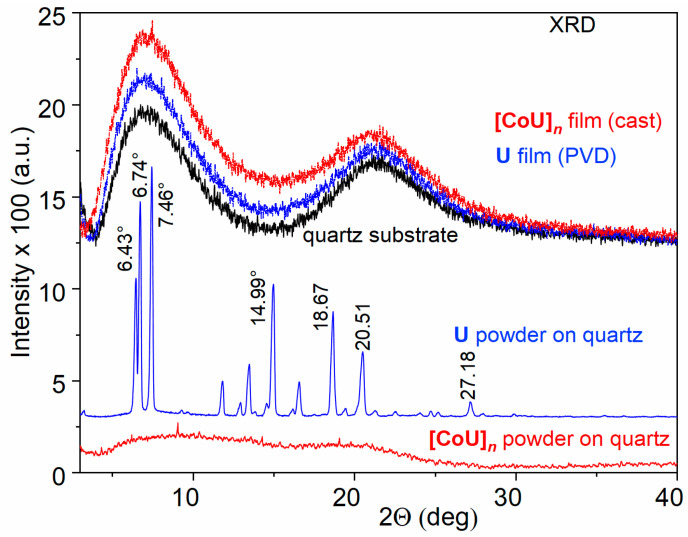
XRD records of powdered materials studied and their films on quartz substrate.

The increased thermal stability of **U** testifies to the presence of relatively strong intermolecular interactions in this material. The powder XRD pattern of the solid unimer **U** (Figure 1) indeed provides evidence for such interactions. The pattern revealed a significant population of regularly spaced X-ray scattering centers in this material, i.e., a significant crystallinity of **U**. The most prominent peaks observed at 7.46°, 6.74°, 6.43°, 14.99°, 18.67°, and 20.51° correspond to d-spacings of 11.8 Å, 13.1 Å, 13.7 Å, 5.9 Å, 4.75 Å, and 4.33 Å, respectively. A small peak at 27.18° could be assigned to π–π stacking interactions of aromatic rings with a distance of 3.3 Å [32,33]. The rigid planar tricyclic carbazole group present in the center of **U** molecules, which enables their π–π stacking and supports their multiple and thus more permanent intermolecular interactions, is most likely responsible for the high thermal stability of **U** that was found.

Unlike unimer **U**, the powdered polymer **[CoU]_n_** exhibited mostly an amorphous structure manifested by two wide humps around 9.1° and 19.7°. The presence of only a few small weak peaks at 4.31°, 9.12°, 14.24°, and 19.70° (d-spacing 20.4 Å, 9.69 Å, 6.21 Å and 4.5 Å, respectively) indicated a very weak organization of the molecules in this material. This is not surprising, because in octahedral [*-tpy-*Co-*tpy-*] linkages, the planes of *tpy* groups are perpendicular to each other. Due to the overlapping of the π-orbitals of the *tpy* groups and the central carbazole unit, the planes of the adjacent **U** units will form angles close to 90°, so that the **[CoU]_n_** chains will be considerably twisted chains, which can be classified as “curly” chains.

The diffractograms of the thin films were dominated by the signal of the quartz substrate. The reason for this is that, in the case of the thin films, the experimental setup allowed for the determination of only the distances between the diffraction planes parallel to the substrate surface, and not others. Therefore, according to the XRD patterns, the films appeared to be amorphous, although this was not the case.

The GIWAXS patterns (Figure S10) indicated a preferable orientation of the molecules inside the **U** film parallel to the substrate with the d-spacing centered at about 10–11 Å. A horizontal cut showed two peaks at α_y_ = 6.53° and 12.75° located at the scattering vector ratio ca. 1:2, indicating a periodic structural alignment of molecules in the in-plane direction with a d-spacing of 13.5 Å. The pattern of the **[CoU]_n_** thin film showed a strong background level (Figure S11), which made it impossible to obtain additional information to those obtained from XRD.

**Figure 2:**
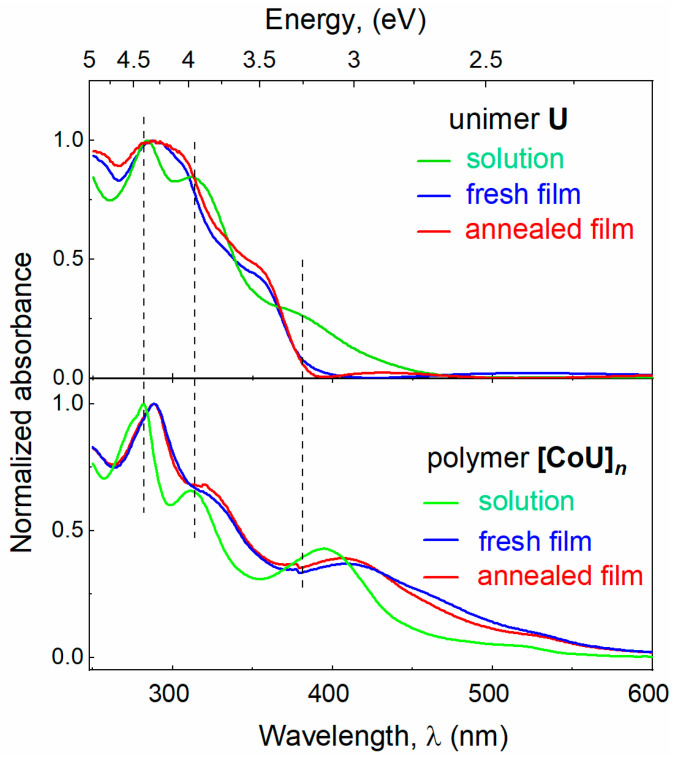
UV–vis absorption and emission spectra (excited at 350 nm) of **U** and **[CoU]_n_**. HFP/ACN (4:1) mixed solvent, film thickness of 300 nm.

Normalized UV/vis absorption and emission spectra of the solutions and thin films of **U** and **[CoU]_n_** are shown in Figure 2 and Figure S12. The absorption bands of the free unimer **U** were generally contributed with π–π* and n–π* transitions in the *tpy* and 9-phenylcarbazole groups [17,34]. The incorporation of **U** molecules into **[CoU]_n_** chains was manifested by a weakening of the absorbance attenuated in the region up to ca. 350 nm and its simultaneous enhancement in the region above this limit. The absorption observed around and above 500 nm was very probably contributed by electronic transitions stemming from the metal-to-ligand charge transfer (MLCT) process [35]. Almost no change in the absorption spectra observed upon thermal annealing points to the stable morphology of the films at elevated temperature.

**Figure 3:**
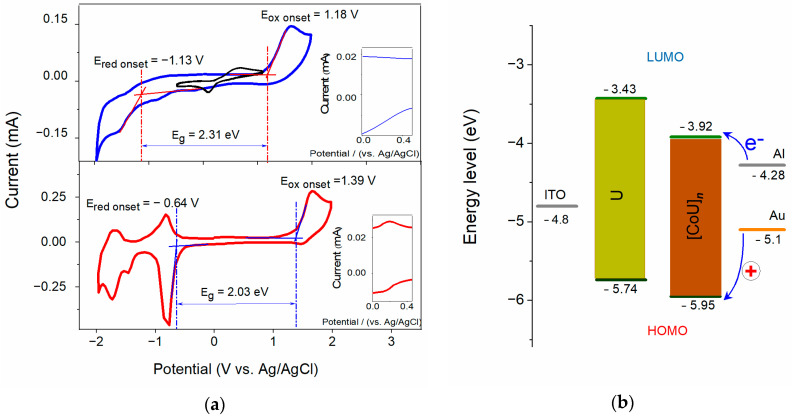
(**a**) Cyclic voltammograms of films (~200 nm) of **U** (blue) and **[CoU]**_n_ (red) deposited on ITO, and ferrocene reference (black top), scan rate 200 mV s^−1^; inset: magnified area around Co^2+^ oxidation potential; (**b**) energy diagrams for sandwich systems with Al and Au top electrodes, ITO bottom electrode, and **U** and **[CoU]_n_** active layers.

Cyclic voltammograms (CVs) for the films (thickness ca. 200 nm) of **U** and **[CoU]_n_** deposited on ITO substrates are shown in Figure 3a. The pronounced oxidation peak with an onset at 1.18 V for **U** and 1.39 V for **[CoU]_n_** can be attributed to the ligand oxidation. The small quasi-reversible oxidation peak observed at approximately +0.20 V, present only in the voltammogram for **[CoU]_n_** (see inset of Figure 3a) and absent in the CV for **U**, was apparently associated with the Co(III)/Co(II) redox pair [36]. The energy of the HOMO orbital (*E*_HOMO_) was calculated according to the following equation [37]:

*E*_HOMO_ = −(*E*_OX_ − *E*_ref_) (1)

where *E*_OX_ is the onset oxidation potential, and *E*_ref_ is the half-wave potential of the ferrocene/ferrocenium external standard system with respect to the Ag/AgCl reference electrode under the same conditions as those of the measured samples, calculated from the average of the maximum and minimum peak potential of ferrocene. The LUMO energy (*E*_LUMO_) was calculated similarly, just using the onset reduction potential *E*_RED_ instead of the oxidation potential. The energy band gap (*E*_g_) was calculated as the difference of *E*_LUMO_ − *E*_HOMO_ between the LUMO energy level (conduction band in inorganic semiconductors) and HOMO level (top edge of the valence band in inorganic semiconductors). The values of the energy levels for both materials are shown in Figure 3b and are listed in Table 1.

### 4.2. Electrical Characteristics

**Figure 4:**
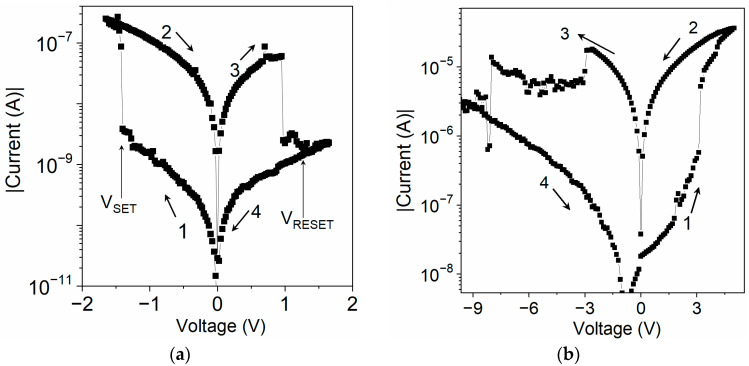
I−V characteristics of sandwich samples: (**a**) ITO/**U** (20 nm)/Al and (**b**) ITO/**[CoU]_n_** (750 nm)/Au. Sweep directions are indicated by arrows. Polarity of applied voltage refers to the top electrode.

The I–V characteristics of the sandwich samples of both materials showed a pronounced hysteresis (Figure 4). A device was initially in a high-resistance OFF-state. When the applied voltage reached the threshold value *V*_SET_, the current increased markedly as the device entered a low-resistance ON-state. It remained in the ON-state, in which the voltage was swept towards the opposite polarity until it reached the *V*_RESET_ value, at which it was reset to the original OFF-state.

The ITO/**U**/Al device showed reproducible bistable switching behavior between high- and low-resistance states with a ratio of currents in the ON- and OFF-states: *I*_ON_/*I*_OFF_ of the order of 10^2^ (Figure 4a), indicating the ability of device to work as an electronic memory. The *V*_SET_ and *V*_RESET_ values showed a dependence on the thickness of the active layer **U**. For example, *V*_SET_ was −1.4 V for a 20 nm layer but less than −3 V for a 200 nm **U** layer. The device with a Ga TE showed electrical properties very similar to those of the Al TE device. However, the Au TE device showed markedly different behavior: its OFF-state resistance was at least two orders of magnitude smaller than that of the Al TE device (Figure S13 in SM). Moreover, the ON-state switch of the Au TE device occurred when the Au was positively biased. In contrast, the Al TE device could not be switched from the OFF-state to the ON-state even with a voltage of over +10 V. This discrepancy can be explained in terms of the values of the HOMO levels of **U**, **[CoU]_n_**, and the work function of ITO and metals, *W*, the negative value of which corresponds to the HOMO level [9]. The negative values of *W*_Ga_ (−4.3 eV) and *W*_Al_ (ca. −4.20 eV) were higher than *W*_ITO_ (−4.8 eV), which was higher than −*W*_Au_ (ca. −5.1 eV). It resulted from the diagram shown in Figure 3b that the energy barrier for the injection of holes from the Au electrode was lower than the one for the injection from ITO. Therefore, hole injection from Au is preferred, whereas electron injection is preferred for both Al and Ga Tes. When the voltage applied to an ITO/U/Au device was reversed to a negative polarity, the device was reset to the OFF-state with nearly the same switching parameters, suggesting that its bistable switching behavior is a material property [38,39].

**Figure 5:**
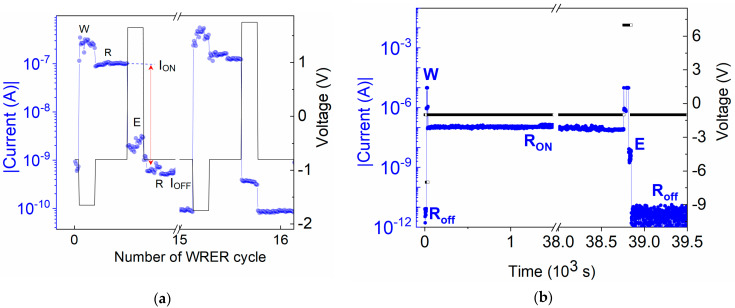
(**a**) I–V characteristics with repeating write, read, erase. and re-read (WRER) pulses and (**b**) persistence of the memory state after one writing pulse at −7 V and erasing pulse at +7 V under continuous reading at −1 V in ITO/**U**/Al device. Blue lines—current, black lines—applied voltage.

The hysteresis in the I–V characteristic indicates that the device can function as an electronic memory. Therefore, tests of dynamic switching during WRER cycles and persistence and volatility tests of the devices were performed (see Figure 5). The WRER test was performed on an ITO/U (20 nm)/Al device using symmetrical switching voltages of V_SET_ = −1.65 V and V_RESET_ = +1.65 V and monitoring the current with a read voltage of −0.8 V for 10 s after each write and erase (Figure 5a). In the persistence test, the current was continuously read at a voltage of −1 V for over 11 h after the device was set to the ON-state (Figure 5b). Persistence after switching the device back to the OFF-state was monitored analogously. The volatility test was performed on an ITO/**U** (200 nm)/Al device with a ten-times-thicker **U** layer (see Figure S14 in SM). The device was first subjected to a voltage sweep from 0 V to −8 V, during which it switched to the ON-state at V_SET_ = −3 V. In the subsequent sweep from −8 V to 0 V, the current followed the ON-state characteristic. The device status was then checked by performing two additional voltage sweep cycles after 5 and 18 h. The state of the device did not change. It remained in the ON-state, even though it was not exposed to an electric field between cycles. In the device used for other I–V characterizations, a relatively shorter retentivity was observed, keeping the retentivity until ~8 h. The reproducible bistable ON and OFF switching together with the remarkable persistence make the active **U** layer device a good candidate for rewritable, non-volatile resistive memory.

Sandwich devices with an active **[CoU]_n_** layer showed similar switching and memory effects to the **U** layer devices but with some differences. As far as switching with positive and negative voltage is concerned, the systems were the same (Figure 4b and Figure S15). However, the **[CoU]_n_** devices showed reduced switching voltages, V_SET_ and V_RESET_, and required a thicker layer to achieve a comparable hysteresis in terms of I–V characteristics (Figure S15). A significant hysteresis was observed only for the devices with layers thicker than 300 nm. Moreover, the reproducibility of the I–V characteristics during cycling and the persistence of the ON-state was worse compared to the **U** devices.

### 4.3. Synaptic Plasticity

A synapse in biological systems is a contact between two adjacent neurons, through which neuronal impulses are transmitted from one neuron to the next one. Upon specific presynaptic neural stimulation, the information is transferred to the postsynaptic neuron in the form of a potential pulse [40,41]. The two terminals of memristors can be correlated to the axon and dendrite of the pre- and postsynaptic neuron, respectively, and the active layer can correspond to the neuronal synapse. A multimodal change in conductance caused by electric impulses can mimic the synaptic weight change [42,43]. The voltage spikes applied on the TE should propagate to the BE through the active layer ideally in a similar way as the action potential generated in the axon of a presynaptic neuron to the dendrite of a postsynaptic neuron via the neuronal synapse. The element formed by the active layer within the TE and BE cross-section could be considered as a single synapse.

In order to prove the neurosynaptic behavior, the prepared memristors were tested with regards to their possible synaptic plasticity, i.e., their resistance, which imitates the synaptic weight, depends on the periodic voltage pulses of different magnitudes and frequencies. The measurements were carried out on both materials under study, with Au or Al or Ga as the TE.

#### 4.3.1. Potentiation and Depression

**Figure 6:**
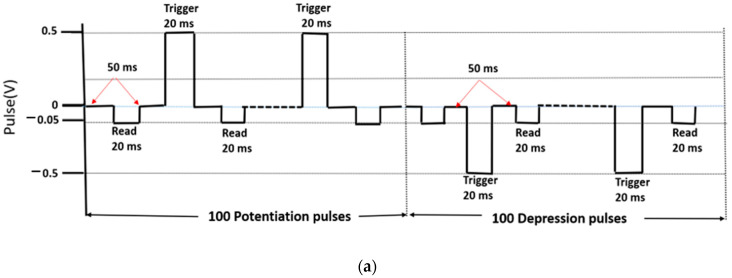

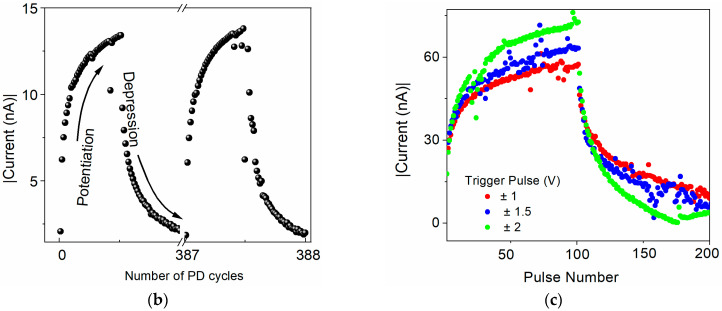
Potentiation and depression in the ITO**/[CoU]_n_**/Au device. (**a**) Measurement scheme of 100 consecutive potentiation pulses followed by 100 successive depression pulses (trigger: ±0.5 V, read: −50 mV). (**b**) Current evolution during the stimulation by the train of trigger pulses and (**c**) current evolution during the stimulation by the train of trigger pulses with different magnitude: ±0.5 V—black symbols, ±1 V—red symbols, ±1.5 V—blue symbols, ±2 V—green symbols. Thickness of the **[CoU]_n_** layer: 200 nm.

In the potentiation and depression studies, the current was expected to continuously increase with the train of input pulses and decrease with train of input pulses of opposite polarity, depending on their magnitude and frequency. The measurement scheme with the timing and polarity of the input pulses applied on the TE is shown in Figure 6a.

As expected from the reproducible I–V characteristics with bistable switching and a very good nonvolatility of the electronic states, the synaptic plasticity was not confirmed in the sandwich devices with **U** as an active layer with any TE material. Although the current increased with the increasing magnitude of the input pulses, it remained almost unchanged when the train of pulses with a fixed magnitude was applied. On the contrary, the devices that employed a **[CoU]_n_** active layer showed a continuous increase in current when exposed to a continuous train of trigger pulses of constant magnitude and in a decrease in current with trigger pulses of opposite polarity (Figure 6b). As can be seen from Figure 6c, the potentiation and depression depended on the magnitude of the trigger pulses. An increased magnitude of the trigger pulses induced enhanced current changes. However, with the absolute value of the magnitude of the trigger pulses being higher than 1.5 V, the current started to decrease/increase back after reaching the saturation with an increasing number of pulses. The potentiation and depression are shown for the device with a Au TE with trigger pulses of ±500 mV and a reading voltage of –50 mV, but it was already observed for trigger pulses of ±200 mV. In the devices with a **[CoU]_n_** active layer, it was also observed when Ga and Al TE materials were used (see Figure S16 in SM). However, with a Au TE, the potentiation and depression were more uniform and reproducible. No significant degradation or alteration in the current level was observed in more than 300 measured cycles, confirming the reproducibility and device stability.

The observed evolution of the current upon the stimulation by trigger pulses clearly mimics the function of a neuronal synapse: the connection is strong with stimulation. In a biological synapse, an increased stimulation with action potential causes a higher discharge of Ca^2+^ ions into the synaptic gap, making the connection between two neurons stronger. After a certain frequency of stimulating events is reached, the connection starts to saturate, illustrating the saturation in learning/memorization and restricting the new process. The similar saturation in the current level observed in our memristive devices demonstrates their analogical functions. Repeated pulses of the reverse polarity bring the device to its original low-conductance state, mimicking the process of depression in bio-synapses.

**Figure 7:**
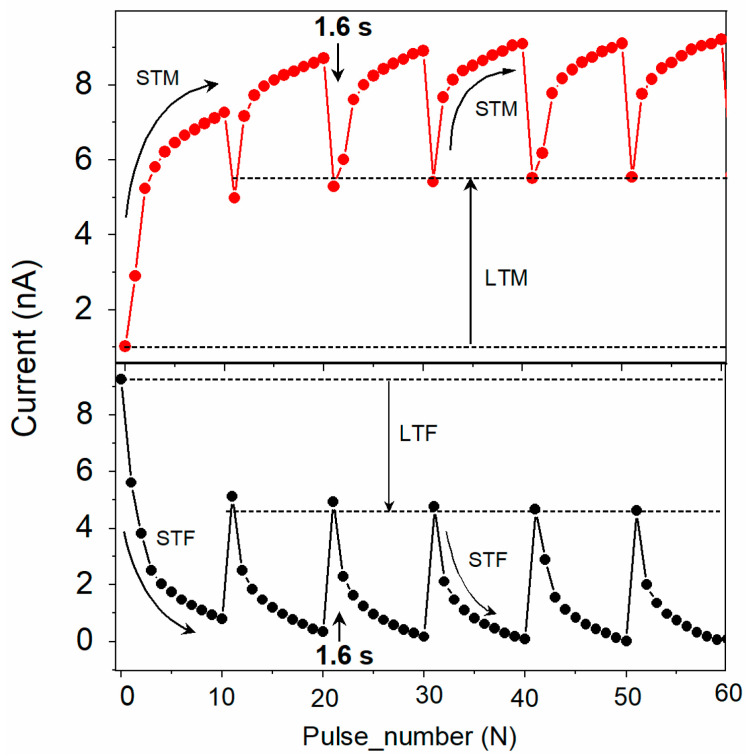
Transition from short-term (STM) to long-term memory (top, red symbols) and short-term to long-term forgetting (bottom, black symbols) for the ITO/**[CoU]_n_**/Au device. One STM cycle consisted of current recorded by ten subsequent trigger pulses (−0.5 V), each followed by a read pulse (±0.05 V). The time gap between subsequent STM cycles was 1.6 s.

In another experiment, six potentiation cycles were obtained using the same measurement scheme as in Figure 6a but introducing a 1.6 s gap between subsequent cycles (Figure 7). With the time gap introduced between cycles, the current dropped down but not fully to the original value (Figure 7 top curve). Repeated cycles with an appropriate time gap stabilized the conductivity, bringing the device memory from short-term memory (STM) to long-term memory (LTM), similar to that described in bio-synapses [5]. A similar transition of short-term forgetting to long-term forgetting (STF to LTF) was observed when the device operated with the reverse polarity (Figure 7 bottom curve).

Such potentiation and depression characteristics in the ITO/**[CoU]_n_**/metal devices demonstrate their potential application in the multimode operations of synaptic learning and neuromorphic computing [42,43]. The memory transition from STM to LTM and from STF to LTF could make the device suitable for studying brain-inspired learning and computing, highly applicable in AI.

#### 4.3.2. Memory Modulation by Paired-Pulse Facilitation (*PPF*) and Depression (*PPD*)

**Figure 8:**
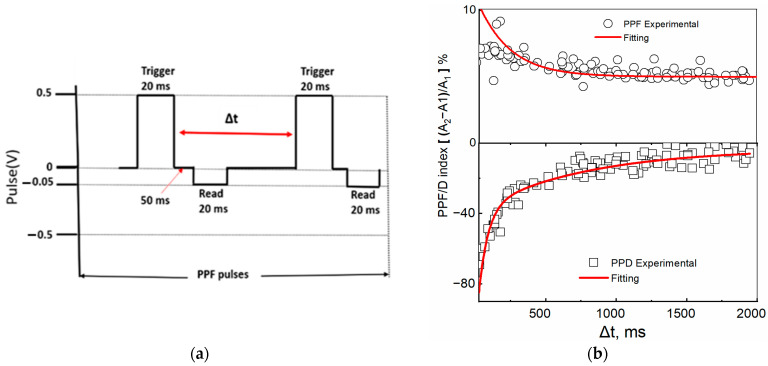
*PPF/PPD* in the ITO/**[CoU]_n_**/Au device. (**a**) A scheme for *PPF* measurement (trigger pulses with opposite polarity but with same magnitude and time scale were used for *PPD*) and (**b**) dependences of the *PPF* (top, circles) and PPD (bottom, squares) indices on the time gap Δ*t* between pulses. Red lines—fitted using Equation (2).

Paired-pulse facilitation/depression (*PPF/D*) refers to the amplification/attenuation of the amplitude of the second of the consecutive excitatory postsynaptic potentials when the synapse is excited by two consecutive presynaptic pulses. It represents a short-term, activity-dependent synaptic plasticity in most chemically transmitting synapses [44,45]. The temporal relation of presynaptic spikes was measured by potentiation/depression modulation in a device with Au and Ga as TE using the set of pulses shown in the scheme shown in Figure 8a. When the current recorded by the reading pulse immediately after the application of the first trigger pulse was indicated by *A*_1_ and the current after the second trigger pulse by *A*_2_, the *PPF/D* index was obtained as the ratio (*A*_2_ − *A*_1_)/*A*_1_. The inter-spike time interval, Δt, varied between 40 ms and 2 s. Shorter time intervals, Δt, induced greater changes in the *PPD*/*F* index than longer Δ*t* intervals. At the shortest interval of Δ*t* = 40 ms, the device showed the following changes in synaptic weight indices: *PPF*~12% and *PPD*~80%. The *PPF/D* indexes were fitted using the double exponential function:

(2) $$PPF/D\;index=C_{0}\pm C_{1}e^{-\frac{\Delta t}{\tau_{1}}}\;\pm \;C_{2}e^{-\frac{\Delta t}{\tau_{2}}}$$

with decay times *τ*_1_, *τ*_2_ and C_0_, C_1_, and C_2_ constants, with C_0_ indicating the asymptotic baseline value. For the ITO/**[CoU]_n_**/Au device, the *PPF* decay times of *τ*_1_ = 0.15 ms and *τ*_2_ = 300 ms and the *PPD* decay times of *τ*_1_ = 65 ms and *τ*_2_ = 780 ms were found. Similar values were observed for the device with a Ga TE (Figure S17), although the initial conductivity was different. In a biological synapse, the facilitation and depression are divided into two time scales: one activating in a rapid phase, lasting only up to tens of milliseconds (*τ*_1_), and the second exhibiting a slower activity, extending to hundreds of milliseconds [46,47]. The values obtained for our system were comparable to those for biological synapses. Similar results were reported by Shin S. et al. [48] for a memristor with an active layer made of a ruthenium-complex-based organic compound with a mixed ionic–electronic conductivity, although, in this case, a high concentration of a salt was needed to observe the PPF phenomena, which suggests that the presence of ions plays an important role in the memristor’s synaptic plasticity.

#### 4.3.3. Short- and Long-Term Memory

**Figure 9:**
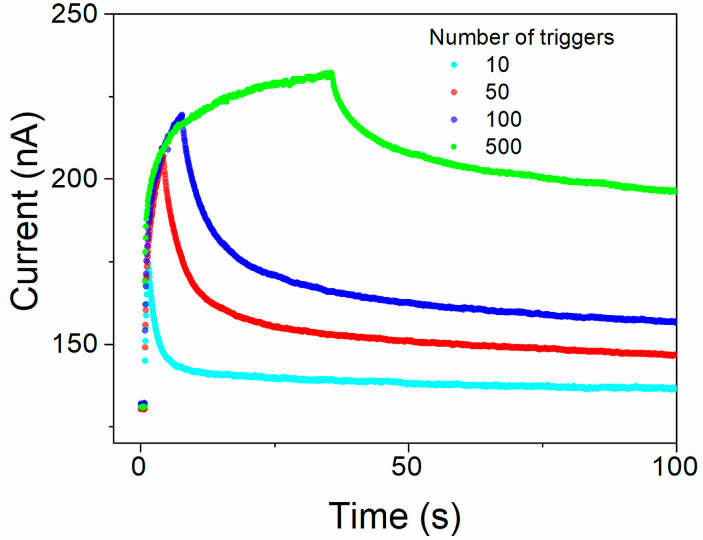
The retention of the memory state in the Au/**[CoU]_n_**/ITO device after 10, 50, 100, and 500 consecutive trigger pulses: background current in the relaxed equilibrium state before triggering, during triggering by multiple pulses (alternating trigger and reading pulses), and during relaxation of the sample after triggering. Read currents recorded using testing voltage pulses 0.05 V with 20 ms duration, excitation trigger pulses + 1 V, and 20 ms pulse duration.

Memory state retention in a device stimulated by different numbers (from 10 to 500) of trigger pulses (+1 V for 20 ms) was measured as the read currents induced by test voltage pulses (0.05 V, 20 ms). Complete measurement cycles consisting of the background current in the relaxed steady state before triggering, with multiple pulses during triggering (alternating trigger and read pulses), and during relaxation of the sample after triggering are shown in Figure 9.

The time courses of the read current decay after the subtraction of the current in equilibrium, without excitation, are shown in Figure S18 for 10, 50, 100, and 500 consecutive trigger pulses, respectively. Similarly to that described previously in the literature [49,50], a stretched exponential function must be employed to satisfactorily describe the process. As can be seen from the inset of Figure S18 showing the decays for 100 and 500 trigger pulses at the extended period of time, there are two processes clearly distinguished, running in different timescales, with the slower one extended to thousands of seconds. The decays could be well fitted by the sum of two Kohlrausch stretched-exponential functions (Equation (3)).

(3) $$I=C_{1}\exp(-{\left(\frac{t}{\tau_{K1}}\right)}^{\beta_{1}})+C_{2}\exp(-{\left(\frac{t}{\tau_{K2}}\right)}^{\beta_{2}})+I_{\infty }$$

where $\tau_{Ki}$ are the decay time constants, $0\leq \;\beta_{i}\leq \;1$ are the stretching exponents, and $I_{\infty }\;$is the current in the non-excited in equilibrium state. The stretched-exponential decay is typical for processes with a wide distribution of rate constants. It can be better characterized by the mean decay time $\left\langle \tau \right\rangle$ of the process that can be obtained as

(4) $$\left\langle \tau \right\rangle =\int_{0}^{\infty }e^{-{\left(\frac{t}{\tau_{K}}\right)}^{\beta }}dt=\left(\frac{\tau_{K}}{\beta }\right)\Gamma \left(\frac{1}{\beta }\right)$$

where Γ is gamma function and *τ_K_* is the time constant obtained from the stretched-exponential (Kohlrausch) Equation (3).

The values of the stretching exponents and the mean decay constants obtained from fitting the experimental data are shown in Table S1 in the Supplementary Materials. Although the obtained parameters were rather ambiguous due to many fitting parameters and relatively noisy data, two process can be clearly distinguished: the faster one, closer to a single exponential decay, with mean decay time constants in seconds and stretching exponents between 0.55 and 1.0 depending on the number of trigger pulses, and the slower process with a stretching exponent β~0.2 and the mean decay time constants exceeding 10^4^ s, less dependent on the number of trigger pulses. Since the **[CoU]_n_** layers contain π-conjugated unimers with counter anions, the conductivity can be affected by doping. The slow decay process can be hence explained by the diffusion-controlled changes in the doping level [51,52,53]. The time scale is within the range of the values reported in the literature for two-terminal devices [50]. The two processes with the markedly different decay constants can be assigned to short-term and long-term memory behavior, respectively [53].

### 4.4. Analysis of the Resistive Changes in U

**Figure 10:**
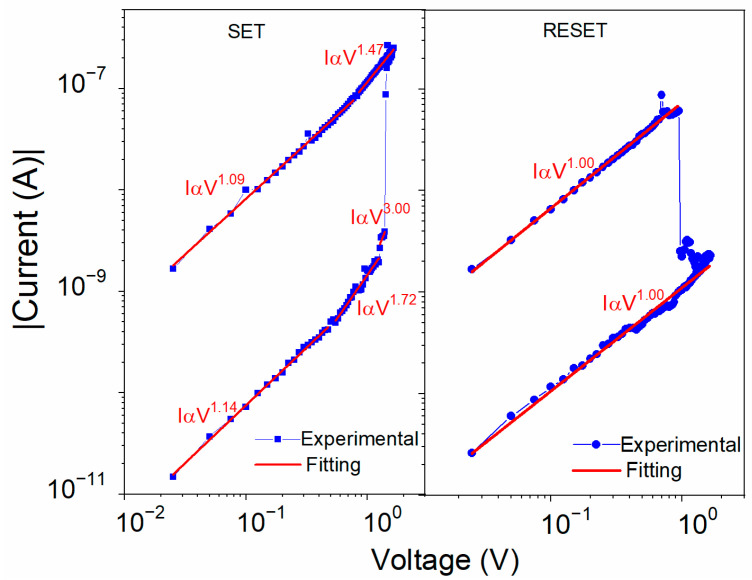
Bi-logarithmic I–V plots for Al/**U**/ITO device during SET and RESET sweeps fitted using the SCLC and ohmic conduction models (red lines).

In order to explain the mechanism of the voltage-induced resistive changes, several phenomena were considered with the respective supporting analysis. The I–V characteristics during the setting and resetting sweeps for the ITO/**U**(20 nm)/Al device are plotted in a log–log scale in Figure 10. The slopes of all the curves showed an almost linear behavior. Only around the *V*_SET_ voltage were steeper slopes observed that could show an influence of the space charge. There were similar energy barriers between the **U** and both electrodes; 0.94 eV for ITO and slightly smaller, 0.85 eV, for Al (Figure 3b). For the interface with the Al electrode, electron injection was slightly more favorable due to the smaller Schottky barrier between the work function of Al and the LUMO level of U. The unit slope of the log I–log V dependence in the low-resistance region (Figure 10) indicates the ohmic conductivity in accordance with the following equation [54]:

(5) $$J\propto V\;exp\left(-\frac{{\Delta E}_{a}}{kT}\right)$$

where ${\Delta E}_{a}$ is the electron activation energy, V is the electric field, k is the Boltzmann constant, and T is the temperature. When the voltage approached *V*_SET_ in the OFF-state, the slope increased firstly to 2 and then to 3. A slope of 2 is typical for the space-charge-limited current (SCLC) in trap-free conditions or in a semiconductor with shallow traps, which can be described by Child’s law [54]:

(6) $$J=\frac{9}{8}\varepsilon_{0}\varepsilon_{r}µ(V^{2}/d^{3})$$

where $\varepsilon_{r}\;$is the relative permittivity of **U**, ε_0_ is the permittivity of free space, μ is the effective charge carrier mobility that also counts for the shallow traps, and *d* is the thickness of the active layer. The steep increase at the *V*_SET_ voltage could be explained by shifting the quasi-Fermi level above the energy level of traps that become filled and no longer influence the charge transport, switching the device to its ON-state [55,56]. The opposite polarity of voltage removes the charges from the localized trapping states, resetting the device to its OFF-state.

## 5. Discussion

Several mechanisms have been proposed for the memristor function of organic molecules. These are primarily intra- and intermolecular charge transfers, the formation of a charge transport complex (CT), voltage-induced changes in the molecular conformation, and the redox and electrode phenomena [9,20,21]. The intra- and intermolecular charge transfer mechanisms are mainly considered for systems with conjugated D-A molecules, which is the case of the unimer **U**, where the central *N*-phenylcarbazole unit (Cbz) is an electron donor to the *tpy* end-groups. Thus, the **U** molecule is an A-D-A type system [57,58,59]. The applied slow and controlled PVD deposition process of **U** molecules onto substrates enables their energetically favored packing to supramolecular structures with reduced interplanar distances. Such morphology should promote effective intermolecular interactions and a uniformity of charge transfer in the **U** film, thereby prolonging the persistence of corresponding devices [14]. The A-D-A characteristic of **U** molecules, in addition, reduces steric hindrances caused by the Cbz group. A shift of electrons from the n-orbital of the nitrogen atom to tpy groups changes the trigonal pyramidal geometry of this center to an almost planar one (it reduces the height of the pyramid) [12]. This enables the packing of **U** molecules by the Cbz|tpy π–π stacking into [D-A] system that enables an efficient inter-molecular D-A charge transfer [13,14,15]. In a **U** molecule, the Cbz–tpy dipoles form an angle of about 100°, giving a resultant intramolecular dipole that interacts with the applied voltage [13,14].

It has been reported that once the charge is injected into the donor unit, it is transferred to the acceptor moiety, creating some charge redistribution in the molecule. This redistribution creates the temporary dipole formation via molecular conformation that can alter the device conductivity [13,14,15]. Q.F. Gu et al. [38] demonstrated a two-state or three-state conductivity and memory effect on a D-A-D system with a diphenyl-sulfone-bridged oxadiazole acceptor, in which the effect was dependent on their dipole moments. Reversing the polarity pulled back the charges from the acceptor to the donor and the device reset to its original state [39].

The unimer **U** might also undergo a voltage-induced polarization effect, leading to a stronger intermolecular interaction between adjacent molecules. Bridging two moieties by an effectively conjugated block can induce a critical change in the molecular reorganization and electron density distribution in the conjugated backbone, bringing an improved memory performance [39].

The I–V analysis of the ITO/**U**/Al device showed the presence of a space charge in the device set to the ON-state. The trapping/detrapping process can be influenced by the dipole reorganizations at the injection barriers [55].

Unlike the unimer **U**, in **[CoU]_n_**, both electronic and ionic conductivity can play a significant role in the memristive switching [9]. The reduction in the energy bandgap of the material (Figure 3, Table 1), as well as reduction in the injection barrier, facilitates the injection of charge carriers. Both the ligand and the Co(II) ion are redox-active, and the diffusion of the counterions can play a role in the changes in the continuous resistance of the device, which are responsible for its synaptic plasticity [60]. The ionic diffusion model was used, for example, by Goswami S. et al. to explain the hysteresis of I–V characteristics in a similar system [9]. There are other mechanisms that could explain resistive memory and synaptic plasticity that are still under study [9,42,43,61].

In the synaptic plasticity studies of devices with a **[CoU]_n_** active layer, the current increased/decreased with the increasing number of trigger pulses until it reached a saturation value similar to “learning restricting new learning” in biological synapses [41,42,43]. Paired pulse facilitation and depression are two functions of biological synapses; the former one occurs as a rapid phase, lasting tens of milliseconds, and the latter is a slower phase lasting hundreds of milliseconds. The values of the time constants that were achieved for the device with a Au and Ga TE were comparable to those measured in biological synapses (Figure 8b and Figure S17) [43,48]. In addition, the observed memory transitions from STM to LTM and from STF to LTF (Figure 7) can allow these memristors to be used for studying brain-inspired neuromorphic systems [5].

## 6. Conclusions

Sandwich structures, composed of an ITO bottom electrode, an Al or Au or Ga top electrode, and a unimer **U** active layer, exhibited non-volatile bistable memory behavior with an ON/OF ratio of over 100, a retention of 18 h, and persistence of 11 h. The conductivity of unimer **U** is mostly dominated by electronic process and supported with supramolecular interactions. In contrast, analogous sandwich structures with **[CoU]_n_** active layers exhibited multimodal resistance changes and synaptic plasticity, mimicking biological neuronal synapses. The reduction in the HOMO and LUMO energy levels and their simultaneous convergence, i.e., the reduction in the bandgap energy, accompanying the self-assembly of the U unimer with Co^2+^ ions to the polymer **[CoU]_n_** is indisputably responsible for the observed substantial difference in the behavior of the mentioned sandwich structures. Another source of the difference in the behavior of the mentioned sandwich structures is the fact that the conductivity of **[CoU]_n_** is contributed not only by electronic processes but also by ion transport. Of the tested ITO**/[CoU]_n_**/TE sandwich structures, the ITO**/[CoU]_n_**/Au sandwich structure appeared to be the best so far, showing synaptic weight changes of around 12% for PPF and 80% for PPD, and their exponential decay was in the time scale of neurosynapses. These properties justify the device’s ability to learn and memorize and offer potential applications of these systems in neuromorphic computation and brain-inspired synaptic devices.

## Figures and Tables

**Table 1:** Energy levels and band gaps of **U** and **[CoU]_n_**.

Compound	*E*_LUMO_ (eV)	*E*_HOMO_ (eV)	*E*_g_ (eV)
**U**	−3.43	−5.74	2.31
**[CoU]_n_**	−3.92	−5.95	2.03

## Data Availability

The raw and processed data required to reproduce these findings are available on request.

## Supplementary Materials

The following supporting information can be downloaded at: https://www.mdpi.com/article/10.3390/polym16040542/s1: Figure S1: MALDI mass spectrum of unimer **U**; Figure S2: NMR spectra of unimer **U** (1H; COSY; 13C) and their assignment; Figure S3: Raman spectra of powdered **U** and **U** film deposited on FTO; Figure S4: FTIR spectra of unimer **U** and polymer **[CoU]_n_**; Figure S5: Surface profiles of the PVD deposited **U** film and the solution cast **[CoU]_n_** film; Figure S6: Surface profile of the device; Figure S7: Physical image of the ITO/**U**/Al device; Figure S8: TGA of the unimer **U** and **[CoU]_n_**; Figure S9: Absorption and emission spectroscopy of **U**; Figure S10: 2D GIWAXS images of **U** thin film on silicon oxide; Figure S11: 2D GIWAXS images of **[CoU]_n_** thin film on silicon oxide; Figure S12: UV-vis absorption and emission spectra of **[CoU]_n_** solution with different concentrations of cobalt salt; Figure S13: I-V characteristics of the ITO/**U**/Au device; Figure S14: Volatility test of the ITO/**U** (200 nm)/Al resistive memory device; Figure S15: I-V characteristics of the ITO/**[CoU]_n_** (300 nm)/Al device; Figure S16: Potentiation and depression in the a) ITO/[CoU] _n_/Ga and b) ITO/**[CoU]_n_**/Al device; Figure S17: PPF/PPD in the ITO/**[CoU]_n_**/Ga device; Figure S18: Long-term decay after excitation of the ITO/**[CoU]_n_**/Au device with multiple trigger pulse. Ref. [62] is cited in Supplementary material.
