# Supplementary material for: Synapse-Mimicking Memristors Based on 3,6-Di(tpy)-9-Phenylcarbazole Unimer and Its Copolymer with Cobalt(II) Ions
Source: Polymers (Basel). 2024 Feb 17;16(4):542. doi: 10.3390/polym16040542 (PMC10892321; doi:10.3390/polym16040542)
Supplement: Supplementary file 1 [file polymers-16-00542-s001.zip › polymers-2828923-supplementary.pdf]

polymers

Supplementary Materials

Synapse-Mimicking Memristors Based on 3,6-Di(*tpy*)-9-Phenylcarbazole Unimer and Its Copolymer with Cobalt(II) Ions

Ambika Pandey [1,2], Andrei Chernyshev [3], Yadu Ram Panthi [1,2], Jiří Zedník [3], Adriana Šturcová [2], Magdalena Konefał [2], Olga Kočková [2], Stephen H. Foulger [4,5], Jiří Vohlídal [3,*] and Jiří Pfleger [2,*]

Table of contents

Figure S1. MALDI mass spectrum of unimer **U.**

Figure S2. NMR spectra of unimer **U** (^{1}H; COSY; ^{13}C) and their assignment.

Figure S3. Raman spectra of powdered **U** and **U** film deposited on FTO.

Figure S4. FTIR spectra of unimer **U** and polymer **[CoU]**$_n$.

Figure S5. Surface profiles of the PVD deposited **U** film and the solution cast **[CoU]**$_n$ film.

Figure S6. Surface profile of the device.

Figure S7. Physical image of the ITO/ **U**/Al device.

Figure S8. TGA of the unimer **U** and **[CoU]**$_n$.

Figure S9. Absorption and emission spectroscopy **of U.**

Figure S10. D GIWAXS images of **U** thin film on silicon oxide.

Figure S11. 2D GIWAXS images of **[CoU]**$_n$ thin film on silicon oxide.

Figure S12. UV-vis absorption and emission spectra of **[CoU]*n*** solution with different concentrations of cobalt salt.

Figure S13. I-V characteristics of the ITO/**U** /Au device.

Figure S14. Volatility test of the ITO/**U** (200 nm) /Al resistive memory device.

Figure S15. I-V characteristics of the ITO/**[CoU]*n*** (300 nm) /Al device.

Figure S16. Potentiation and depression in the a) ITO/**[CoU]*n*** /Ga and b) ITO/**[CoU]*n*** / Al device.

Figure S17. PPF/PPD in the ITO/**[CoU]**$_n$/Ga device.

Figure S18. Long-term decay after excitation of the ITO/[CoU]$_n$/Au device with multiple trigger pulses.

1. MALDI mass spectrum

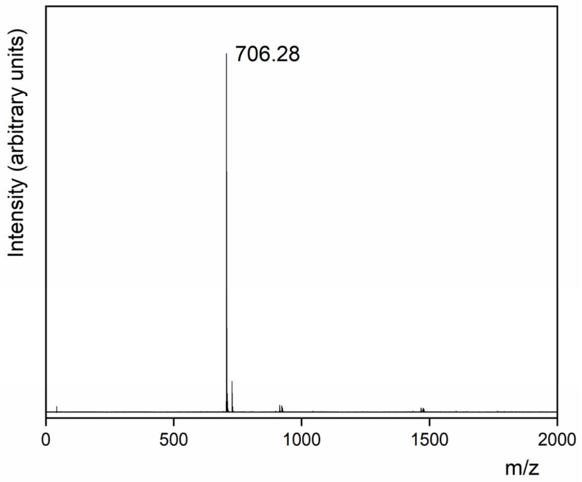

Figure S1. MALDI mass spectrum of unimer **U.**

Found m/z: 706.28; theory for $C_{48}H_{31}N_7$: 706.27. Procedure: First, a matrix layer from an aqueous solution of 2,5-dihydroxybenzoic acid (1 L; 20 mg mL^{-1}) was cast onto a ground steel target and allowed to dry. A layer of unimer from its solution in HFP/ACN (4:1) was then applied to the matrix layer and allowed to dry.

2. NMR spectra of unimer U (^{1}H NMR; COSY; ^{13}C NMR) and their assignment. (Figures S2a–c)

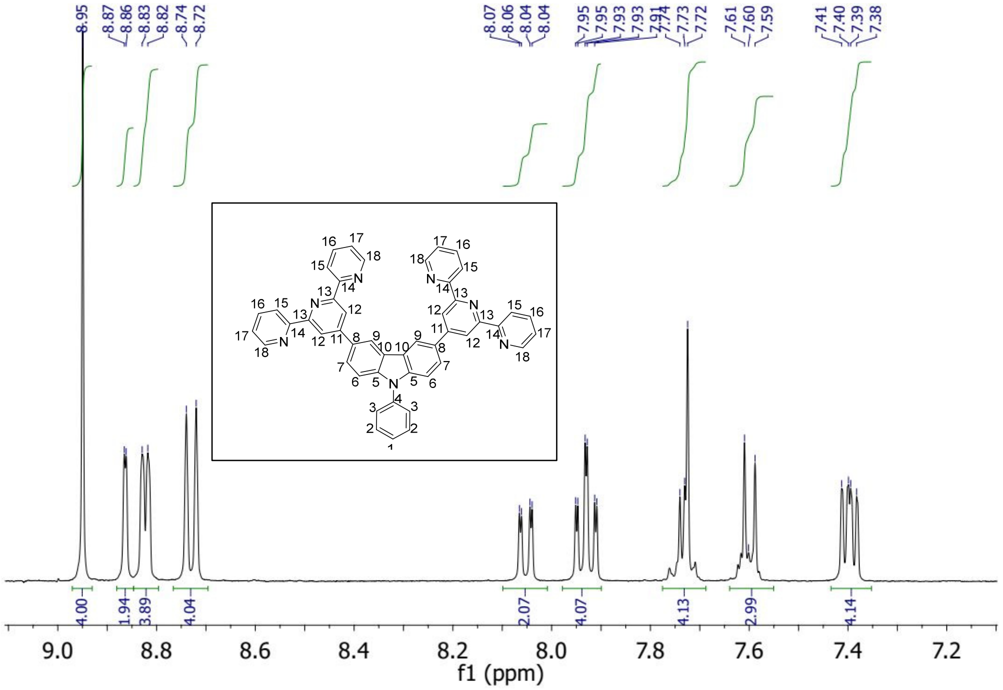

^{1}H NMR (400 MHz, deuterated 1,1,2,2-tetrachloroethane,TCE-d_2, 110°C) δ 8.95 (s, 4H^{12}), 8.87 (s, 2H^9), 8.82 (d, J = 4.7 Hz, 4H^{15}), 8.73 (d, J = 7.9 Hz, 4H^{18}), 8.05 (dd, J_1 = 8.6 Hz, J_2 = 1.8 Hz, 2H^3), 7.93 (td, J_1 = 7.7 Hz, J_2 = 1.8 Hz, 4H^{16}), 7.73 (m, 4H, H^6 + H^7), 7.61 (m, 3H, H^1 + H^2), 7.40 (dd, J_1 = 7.0 Hz, J_2 = 5.3 Hz, 4H^{17}).

Figure S2. ***a*:** ^{1}H **NMR spectrum** of unimer **U** and its assignment.

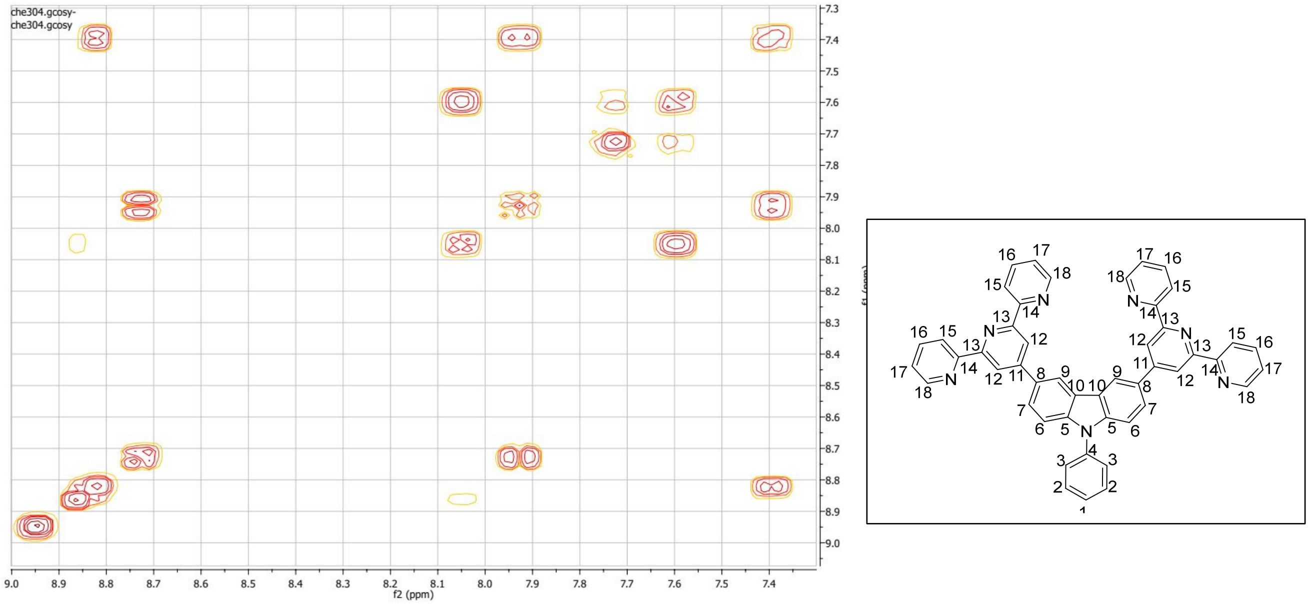

Figure S2. ***b:*** ^{1}H COSY NMR spectrum of unimer **U**.

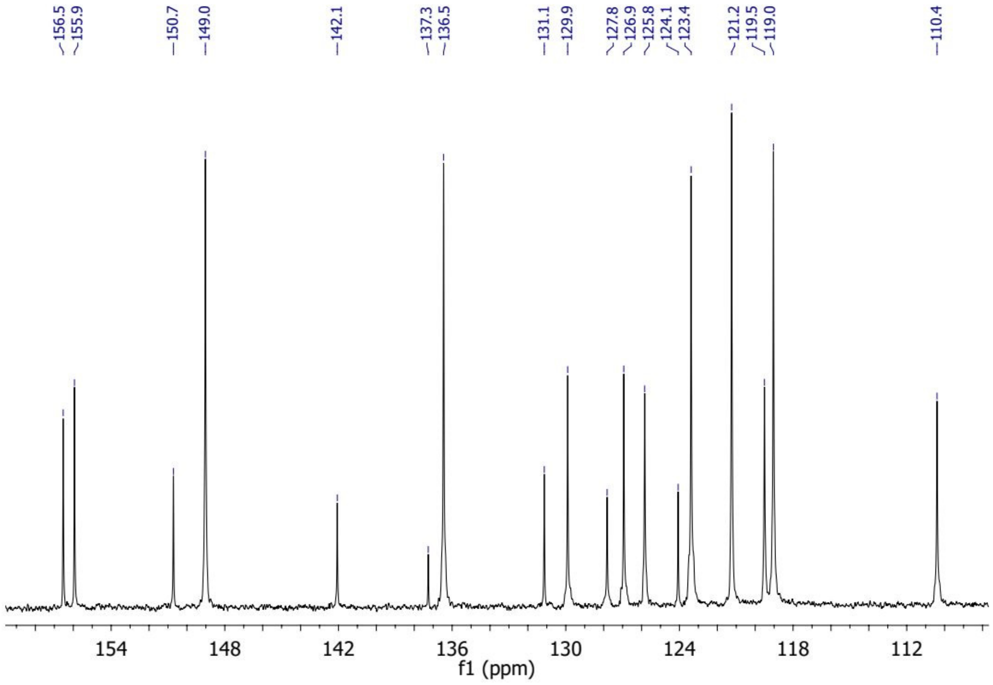

^{13}C NMR (151 MHz, TCE-d_2, 110°C) δ 156.5 C^{13}, 155.9 C^{14}, 150.7 C^{11}, 149.0 C^{18}, 142.1 C^{8}, 137.3 C^{5}, 136.5 C^{16}, 131.1 C^{4}, 129.9 C^{2}, 127.8 C^{3}, 126.9 C^{1}, 125.8 C^{9}, 124.1 C^{10}, 123.4 C^{17}, 121.2 C^{15}, 119.5 C^{7}, 119.0 C^{12}, 110.4 C^{6}.

Figure S2. ***c:*** ^{13}C NMR spectrum of **U** and its assignment.

3. Raman spectra of U - powder and film

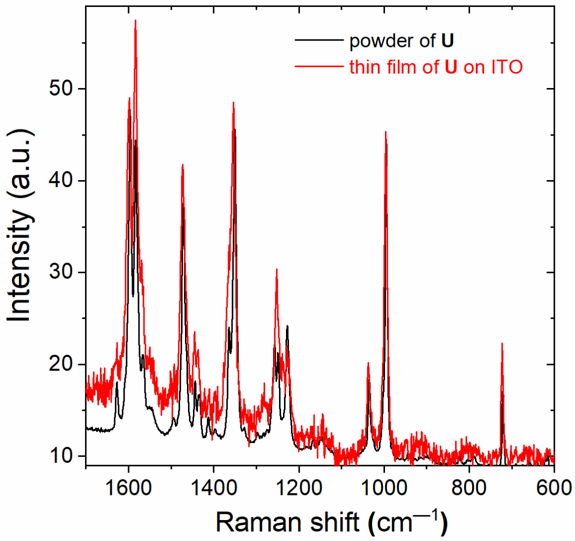

Figure S3. Scaled Raman spectrum of powdered **U** and film of **U** deposited on FTO.

4. Infrared spectra of the unimer U and polymer [CoU]*n*

The IR spectra on powdered and on thin layers deposited on ITO of **U** and **[CoU]*n*** are shown in **Figure S4.** In both compounds, the bands present in the region 1625—1430 cm^{-1} are assigned to the skeletal carbon-carbon and to carbon-nitrogen stretching vibrations, vibrations of both – the polynuclear condensed rings of carbazole and the aromatic heteronuclear pyridyl rings are infrared-active in this region [S1]. The bands in the region 1290—990 cm^{-1} are assigned to the C—H in-plane deformation vibrations, while C—H out-of-plane deformation bands are seen between 900—650 cm^{-1}. There are blue- and red-shifts in the positions of the individual bands and increased linewidths throughout the spectrum of **[CoU]n** when compared to the spectra of **U.** Spectral deconvolution would probably show that the number of bands in the region of skeletal vibrations is the same for both compounds, but the exact band positions, linewidths and relative intensities would most likely be proven not identical. Significant shifts of 10 cm^{-1} – 20 cm^{-1} in band positions, the presence of the two new intense bands at 1175 cm^{-1} and at 1086 cm^{-1}, as well as the change of the band at 881 cm^{-1} in **U** spectrum into a doublet (889 cm^{-1} and 878 cm^{-1}) in **[CoU]n** spectrum most probably stem from Coulombic interactions of tertpyridyl rings with the cobalt ions. It is also likely that there is also increased scattering present in this spectral region causing distortions of the baseline of **[CoU]n** spectrum.

In the GA ATR FTIR spectra of the thin films of both materials deposited on ITO substrates, the band positions of **U** thin layer do not coincide fully with the band positions detected in its powder form. Blue shifts to higher wavenumbers of about 5 cm^{-1} are observed for the skeletal carbon-carbon stretching bands, while a red shift of about 5 cm^{-1} to lower wavenumbers is observed for the band originally located at 881 cm^{-1} in the bulk spectrum. The relative blue shift of the skeletal C—C stretching bands and of the in-plane deformation bands in GA ATR spectra of thin layers might be an artefact since, generally, the ATR spectra have bands shifted to lower wavenumber compared to transmission spectra and two types of ATR method were used to obtain the two (powder and thin layer) spectra. If this relative blue shift was a real effect, it would indicate a larger force constants in thin layer molecules that could be induced by the molecules' altered and stiffer conformation. The red shift observed in the thin layer of **U** compared to its bulk powder form for the out-of-plane deformation band originally at 881 cm^{-1} in the **U** powder is most likely a real effect and not an artefact since the band at the higher and lower wavenumbers are shifted in the opposite direction (to the blue) in the thin layers. This red shift indicates,

that the angles involved in the respective deformation vibration are more easily deformable pointing to alterations in the molecules' conformation. The band that occurs at 769 cm^{-1} in the powder spectrum is most likely shifted to significantly smaller wavenumbers (to about 740 cm^{-1}) and is greatly increased in intensity (about two-fold for **U** deposited on ITO) since there is no band detected at the original position of 769 cm^{-1} nor nearby. Such spectral behavior is most likely a result of changes in the transition dipole moment for the respective vibrations in **U** that might be caused by new intermolecular interactions between **U** molecules and the ITO substrate.

When **[CoU]*n*** is cast (*via* solution drop casting) onto the ITO electrode to form thin layers, there are no significant wavenumber shifts in the skeletal carbon-carbon and carbon-nitrogen stretching region (1625 cm^{-1} – 1430 cm^{-1}), but subtle changes in the line widths and in relative intensities especially might be detected in a more through analysis. In the C—H in-plane deformation region (1290 cm^{-1} – 990 cm^{-1}), clearly visible spectral changes are observed: (i) Discontinuities such as bands at 1282 cm^{-1} and at 1175 cm^{-1} in the bulk powder spectrum disappearing or diminishing in thin layer spectra, (ii) bands at 1306 cm^{-1} and at 1239 cm^{-1} increasing in intensity in thin layer spectra compared to the powder, and (iii) the intense and broad band at 1086 cm^{-1} in powder spectrum is blue-shifted to 1090 cm^{-1} in the spectrum the thin film. Following after this spectral region of characteristic vibrations, the 889/878 cm^{-1} doublet becomes a singlet with position only at 878 cm^{-1} in the thin film (a singlet is found in thin film of the original ligand **U** deposited on ITO. The intensity reduction of the strong broad band at 1175 cm^{-1} when transferring **[CoU]*n*** molecules from powder to thin layer might indicate a preferred orientation being adopted by complex **[CoU]*n***, or it might indicate that certain structural forms such as coordination sites, isomers are being excluded or reduced in thin films compared to the powder as a result of highly likely interactions of the complex **[CoU]*n*** molecules with the ITO substrate. Such interpretation is further supported by the observed narrowing, increased symmetry and blue shift of the band at 1086 cm^{-1} in **[CoU]*n*** powder to 1090 cm^{-1} in the thin film. The narrowing of the linewidth of the band at 1086 (1090) cm^{-1} could also be explained not only by a certain preferred orientation or structural form (coordinate sites, isomers) of the complex **[CoU]*n*** molecules being adopted, but it could also imply formation of domains with increased order. These possible differences in the orientation, structure and crystallinity of **[CoU]*n*** in thin fims compared to is powder form are also indicated by the suppressed scattering background of the spectra.

[S1] Socrates, G. *Infrared and Raman Characteristic Group Frequencies: Tables and Charts*, 3rd ed.; Wiley: Hoboken, NJ, USA, 2004. Available online: https://www.wiley.com/en-sg/Infrared+and+Raman+Characteristic+Group+Frequencies%3A+Tables+and+Charts%2C+3rd+Edition-p-9780470093078 (accessed on 16 December 2023).

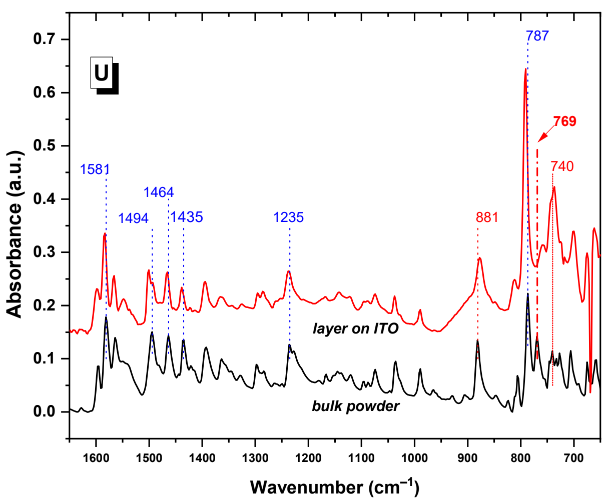

(a)

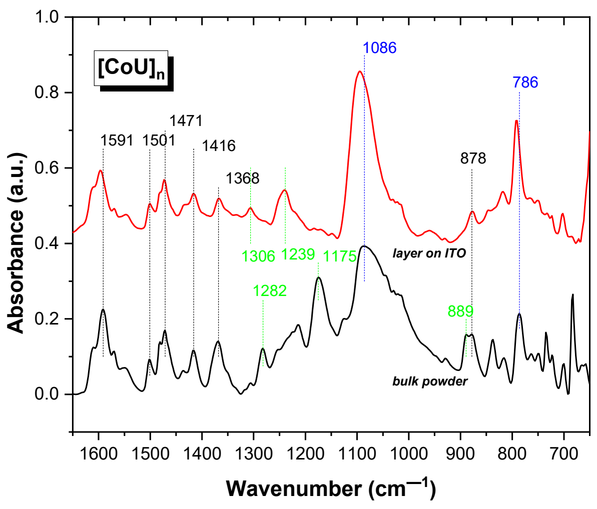

(b)

Figure S4. FTIR spectra of the (a) unimer **U** and (b) polymer **[CoU]**$_n$, black: ATR spectra of powdered samples; red: GA ATR spectra of thin layers deposited on ITO (scaled up to enable comparison).

5. Surface profiles

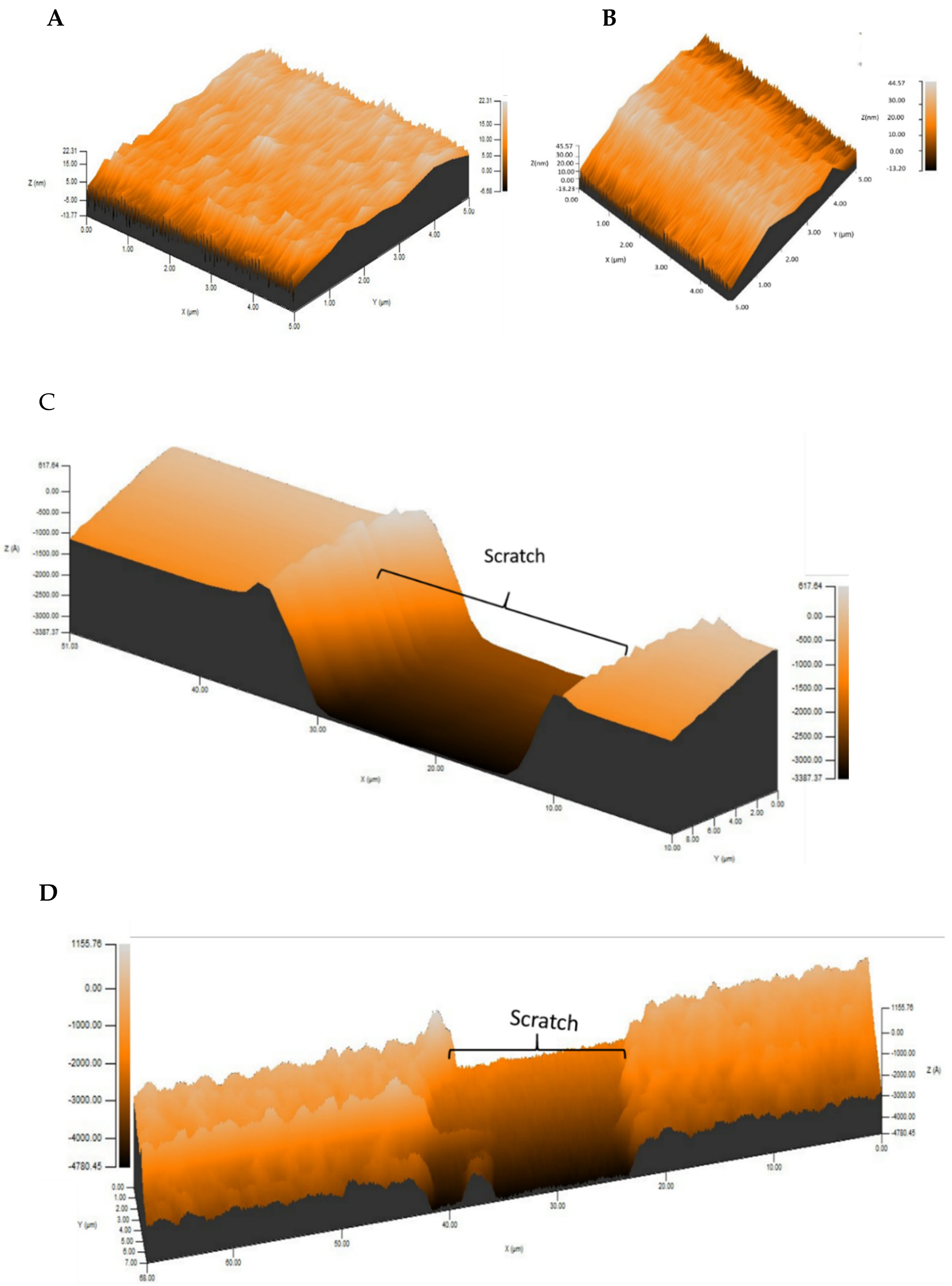

Figure S5. 3D surface of an **U** layer (thickness 300 nm) deposited by PVD on ITO (A and C) and of a solution cast layer (400 nm) of **[CoU]**$_n$ (B and D).

Scans, done by using a Tencor P-17 stylus profilometer (KLA, U.S.A.), were taken from two different areas with a high resolution, scan speed of 2 μm/s, scanning density of

1 line per 500 nm and the bandwidth of 2000 Hz to acquire the highest possible amount of data. No pinholes were observed. Surface scans of **U** and **[CoU]*n***, with two scan areas with, were performed. The mean roughness found was ~10 nm for the PVD cast **U** and ~40 nm for the solution cast **[CoU]*n***.

6. Surface profile of the device

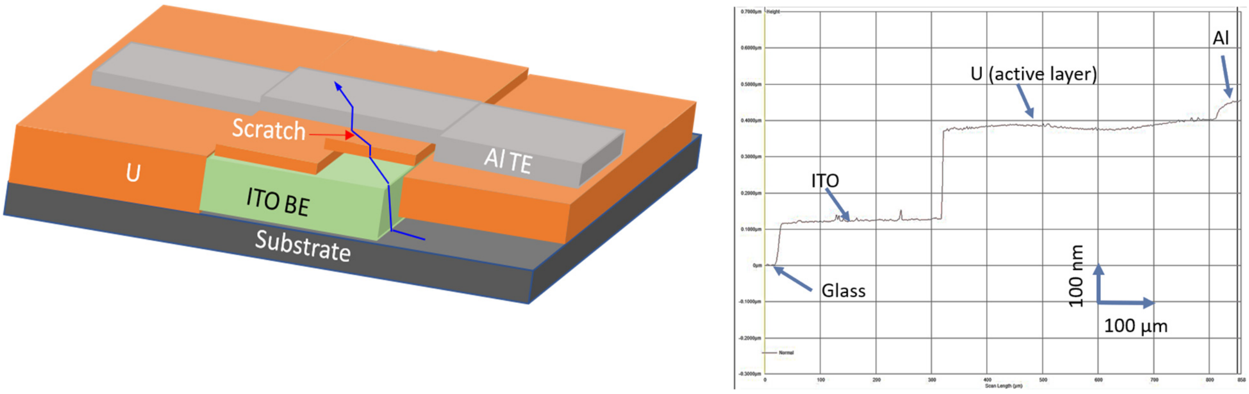

(a) (b)

Figure S6. a) Scheme of the **[CoU]n** device composition with a scratch and a profilometer stylus path (blue), b) device profile as obtained from the profilometer.

7. Physical image of the ITO/ U/Al device

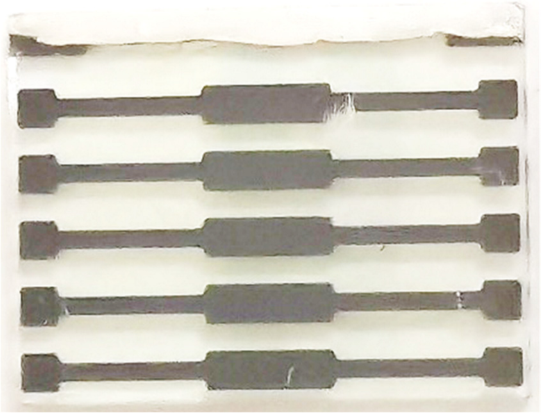

Figure S7. Physical image of the ITO/ U/Al device.

8. Thermal stability of U

The TGA analysis showed the decomposition of the **U** material started at 430 °C and the main decomposition occurred at 470 °C. To further confirm the material's chemical stability with temperature, emission spectra were measured by dissolving the **U** in HFP/ACN before deposition and after PVD by dipping the layer in HFP/ can mixed solvent. The similar spectra revealed no change in the material during during its sublimation in a vacuum (10^{-7} mbar).

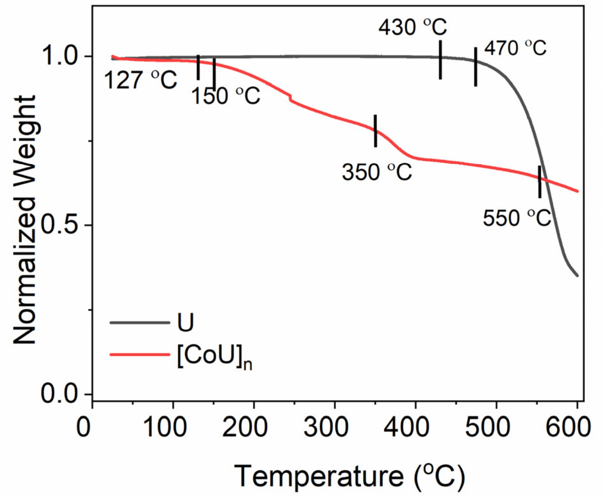

Figure S8. TGA of the unimer **U** (black line), and polymer **[CoU]*n*** (red line). Heating rate 10 °C/min, measured in nitrogen atmosphere.

9. Absorption and emission spectroscopy

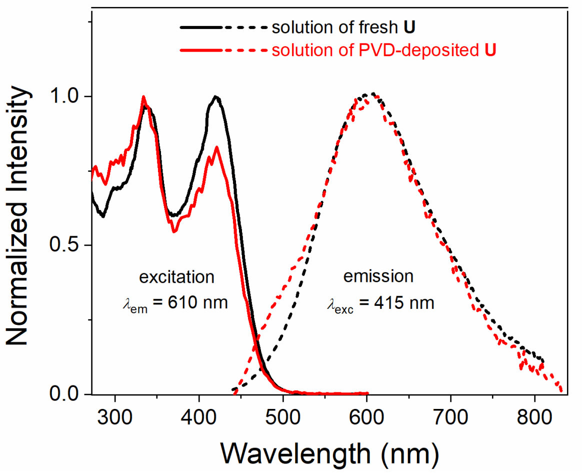

Figure S9. Normalized excitation and emission spectra of the fresh **U** and the PVD deposited **U** dissolved in HFP/ACN (4:1).

10. GIWAXS of U thin film on silicon oxide

The 2D GIWAXS maps obtained for **U** and **[CoU]n** thin films are presented in **Figure S10 and S11**. The lower part of the images with the strongest intensity corresponds to the specular reflection, for which the incident angle is equal to the exit angle. It is observed as a high-intensity spot at α_z = 0.38°. The Yoneda peak, which appears when the exit angle matches the critical angle α_c for the total external reflection of the material, was observed at α_z = 0.40° for **U** and α_z = 0.43° for **[CoU]*n*** films. As the Yoneda peak position is correlated with electron density, the larger value of the Yoneda peak obtained for **[CoU]*n*** denotes the higher density of this thin film. For detailed analysis, the vertical line cuts at α_z direction were performed at the α_y = 0.0° and 0.9° to exclude the specular reflection overlapping with the Yoneda band. Also, the horizontal line cut was performed at the α_y equal to the Yoneda peak position. The intensity was integrated over a width of 5 pixels for each cut to improve the statistics. The intensity profiles versus wave vectors α_y and α_z correspond to the molecular arrangement in in-plane and out-of-plane directions, respectively. In the case of the **U** sample, a weak, arc-shaped signal in the out-of-plane direction is visible on the 2D image (marked with the red arrow), with the center around 8.50°. This indicates that there is a weak preferable orientation of molecules inside the film in the direction

parallel to the substrate, with a distribution of d-spacing centered about 10-11 Å [S2]. Additionally, a weak ring scattering is visible at about α_z = 6.40° (marked with a black arrow). It is also visible as a small peak at the vertical cut (Fig. S10(b)).

The GISAXS results are in agreement with the XRD pattern of **U** film, where also two weak peaks with a similar position are present. Moreover, on a horizontal cut (Fig S10c), two peaks are visible at α_y =6.53° and 12.75°. The peaks are located at the scattering vector ratio ~ 1:2, which indicates a periodic structural alignment of the molecules in the in-plane direction, with a d-spacing of 13.5 Å. The presence of a weak full-ring scattering suggests the polycrystalline nature of the sample, with randomly oriented crystalline domains.

In the case of **[CoU]**$_n$ thin film, on both the 2D image and vertical/horizontal cuts a very high background level is visible due to the emission of cobalt atoms. Because of this, it is impossible to say if any additional peaks are present in the spectra. As such a high background was not visible in XRD diffractograms (Fig.S11 and Fig. 1 in the main text), it is possible that most of the cobalt atoms are localized on or near the sample surface, as the GIWAXS is a very "surface sensitive" method.

[S2] Hou, X.; Xiao, X.; Zhou, Q.H.; Cheng, X.F.; He, J.H.; Xu, Q.F.; Li, H.; Li, N.J.; Chen, D.Y.; Lu, J.M. Surface Engineering to Achieve Organic Ternary Memory with a High Device Yield and Improved Performance. *Chem. Sci.* **2017**, *8*, 2344–2351, doi:10.1039/C6SC03986C

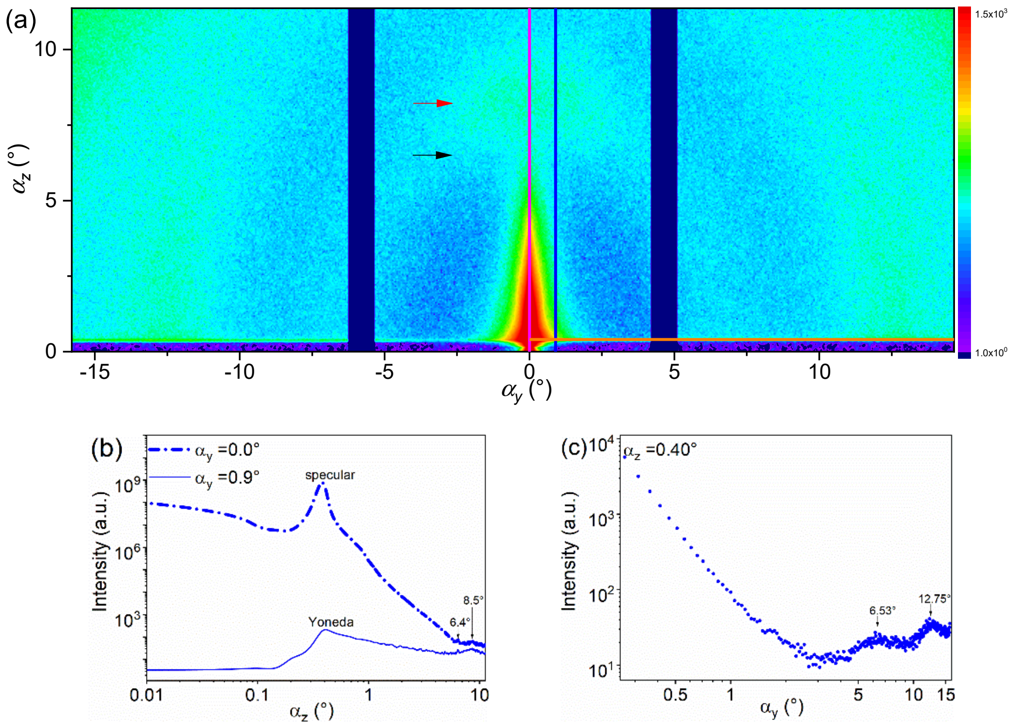

Figure S10. 2D GIWAXS images of U thin film in silicon oxide substrates (a), acquired at α_{inc}=0.19° together with (b) corresponding vertical and (c) horizontal line cuts. Thickness of the layer 300 nm.

11. GIWAX images of [CoU]ₙ thin film on silicon oxide substrates

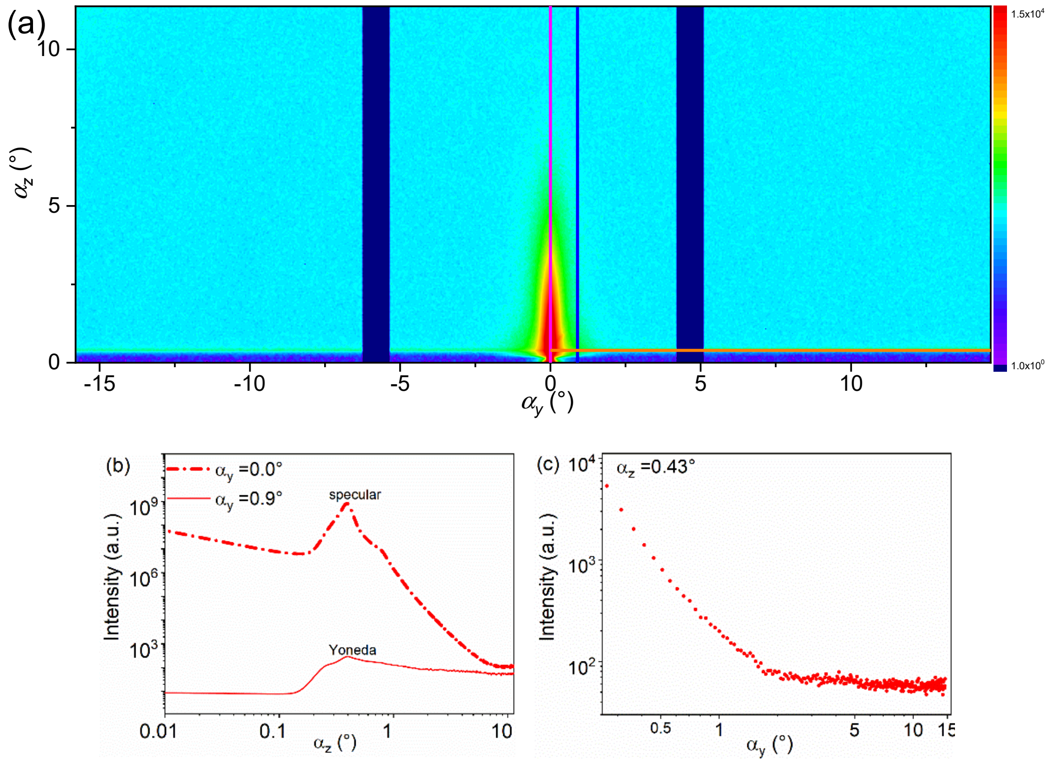

Figure S11. 2D GIWAXS images of **[CoU]*n*** thin film on silicon oxide substrates (a) acquired at α_{inc}=0.19° together with (b) corresponding vertical and (c) horizontal line cuts. Thickness of the layer around 300 nm.

12. UV-vis absorption and emission spectra of [CoU]ₙ solution with different concentrations of cobalt salt

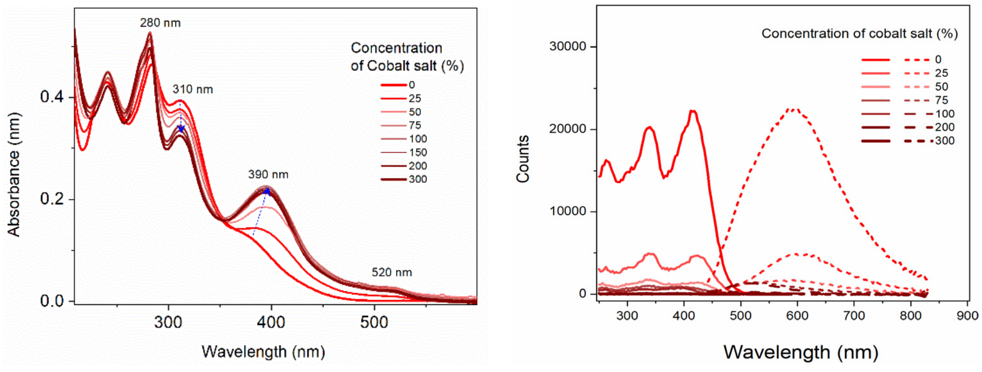

(a) (b)

Figure S12. UV-vis absorption (left) and emission spectra (right) of **[CoU]*n*** in HFP/ACN (4:1) solution with different concentrations of cobalt salt (Concentration: 10^{-5} M). Excitation spectra (solid lines) with a wavelength at 610 nm, and emission spectra (dashed lines) with an excitation wavelength at 420 nm.

The UV-vis spectra of **U** with different ratio of cobalt salt mixing was measured and the spectral evolutions with change in intensity were observed in all peaks at around 280 nm, 310 nm, 390 nm, and 520 nm wavelength. At 280 nm and 390 nm peaks, the increased

ratio of cobalt salt has increased intensity with blue shift and red shift, respectively. The highest shift and intensity change around the 400 nm peak indicates the facilitation of metal-ligand charge transfer with an increased ratio of cobalt-salt supported by increased metal-ligand coordination. A similar finding was observed in fluorescence emission spectra in which the intensity decreased with an increase in salt content both in excitation and emission spectra, indicating the charge transfer as the dominant relaxation phenomenon.

13. I-V characteristics of the ITO/U /Au device

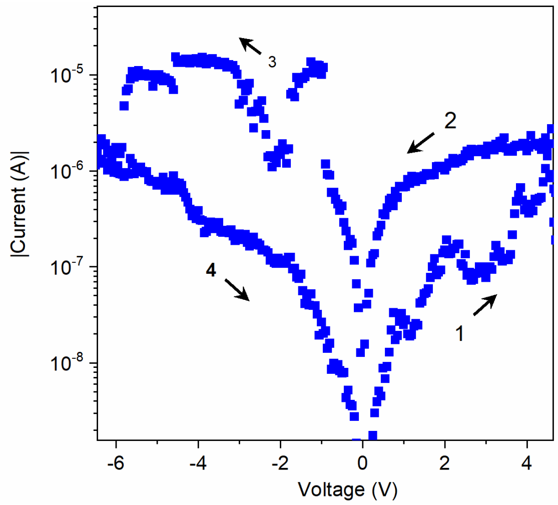

Figure S13. I–V cycles of device ITO/**U** (200 nm) /Au. Sweep directions are indicated by the arrows, device biased through the TE.

14. Volatility test of the ITO/U (200 nm) /Al resistive memory device.

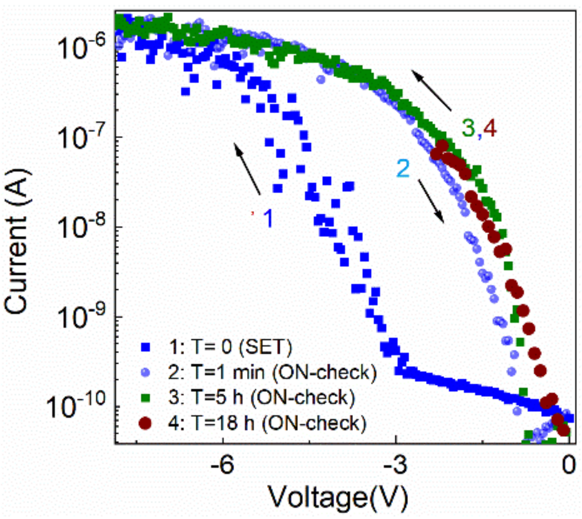

Figure S14. Volatiity test of the resistive ITO/**U** (200 nm) /Al memory device.

15. I–V characteristics of device ITO/[CoU]$_n$ (300 nm) /Au

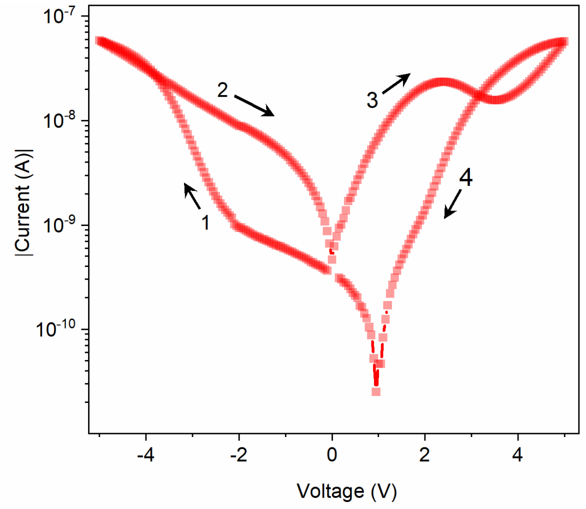

Figure S15. I-V characteristics with resistive memory of ITO/**[CoU]**$_n$ (300 nm) /Al device.

16. Potentiation and depression in the a) ITO/[CoU]$_n$ /Ga and b) ITO/[CoU]$_n$ / Al device

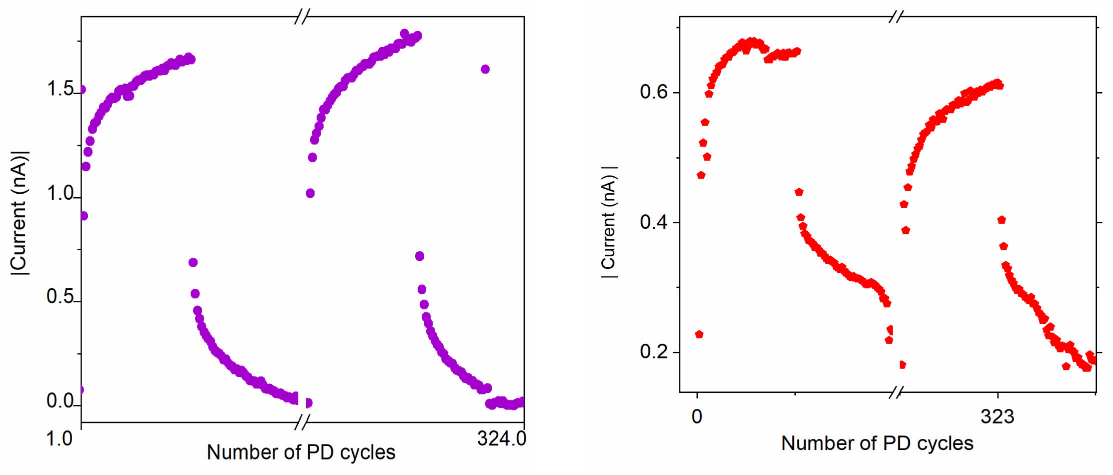

(a) (b)

Figure S16. Potentiation and depression in the a) ITO/**[CoU]**$_n$ /Ga and b) ITO/**[CoU]**$_n$ / Al device. Trigger pulses ±1 V of 20 ms duration, reading at 100 mV for 20 ms, 50 ms rest time between trigger and read pulses (see Fig. 6a for the measurement scheme).

17. PPF/PPD in the ITO/[CoU]n/Ga device

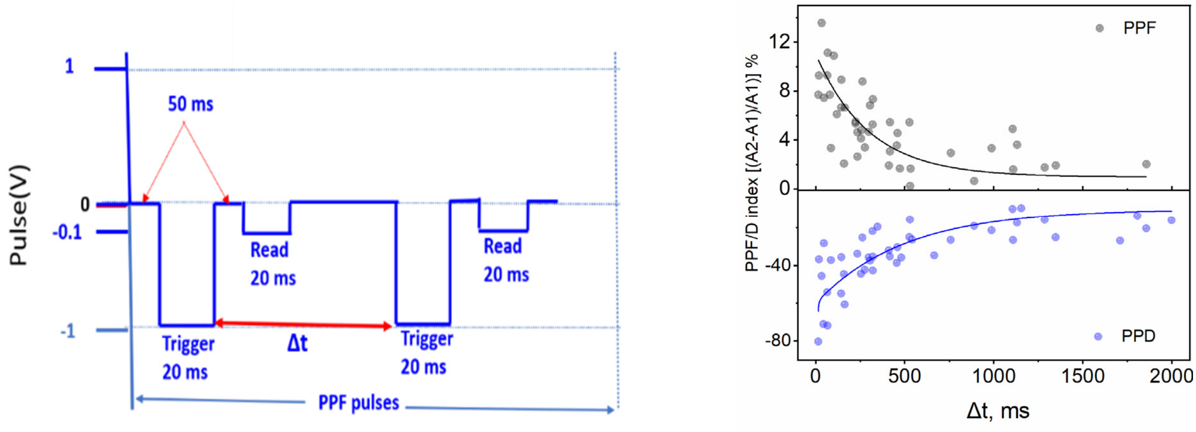

Figure S17. PPF/PPD in the ITO/[CoU]n/Ga device. a) Measurement scheme for PPF measurement (trigger pulses with opposite polarity but with the same magnitude and time scale were used for PPD) and b) PPF (top) and PPD (bottom) index as a dependence on time gap Δt between two pulses.

18. Long-term decay after excitation of the ITO/[CoU]n/Au device with multiple trigger pulses

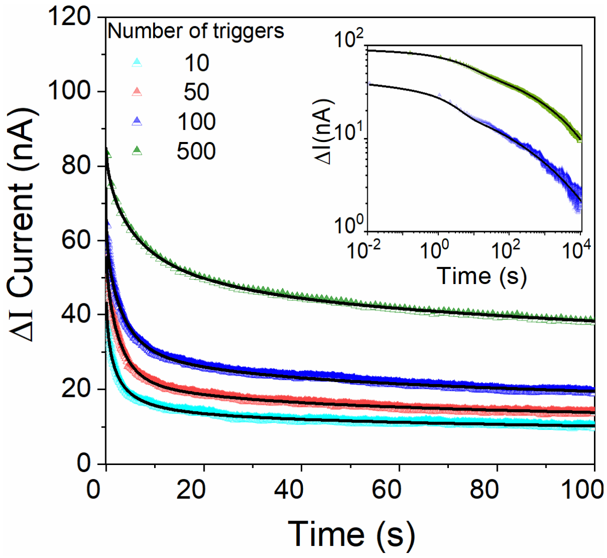

Figure S18. Increment of the read current in the ITO/**[CoU]**n/Au device after the excitation with 10, 50, 100 and 500 concecutive trigger pulses: data fitted using double stretched exponential function (Eq. 3 in the main text). Trigger pulses -1 V, 0.02 s/0.05 s, read pulses -0.05 V, 0.02 s/0.05 s. See Table S1 for fitting parameters.

Table S1. Fitting parameters of current decays using double stretched exponential.

Number of trigger pulses	Weight K_1 [nA]	Stretching exponent β_1	Mean decay time $<\tau_1>$ [s]	Weight K_2 [nA]	Stretching exponent β_2	Mean decay time $<\tau_2>$ [10^4s]
10	30	0.55	2.1	24	0.22	1.1
50	19	1.0	3.2	104	0.10	37
100	8.9	0.98	3.2	41	0.16	1.3
500	21	0.71	9.1	72	0.24	1.7
